# Stress-Induced Metabolic Reprogramming in the Green Microalga *Chlorella* sp. SLA-04

**DOI:** 10.3390/microorganisms14092092

**Published:** 2026-09-18

**Authors:** Calvin L. C. Goemann, Huyen Bui, Sandra Rincon Miranda, Adrienne D. Arnold, Ross P. Carlson, Sridhar Viamajala, Robin Gerlach, Blake Wiedenheft

**Affiliations:** 1 Department of Biological and Environmental Sciences, Carroll College, Helena, MT 59625, USA; 2 Department of Microbiology and Cell Biology, Montana State University, Bozeman, MT 59717, USA; adrienneda@vt.edu; 3 Center for Biofilm Engineering, Montana State University, Bozeman, MT 59717, USA; 4 Department of Chemical and Biological Engineering, Montana State University, Bozeman, MT 59717, USA; 5 Department of Chemical Engineering, University of Toledo, Toledo, OH 43606, USA

**Keywords:** green algae, high-alkalinity, nitrogen depletion, transcriptome, physiological adaptation

## Abstract

Microalgae thrive in diverse and often challenging environments by coordinating metabolic and physiological responses to environmental stress, yet the molecular mechanisms underlying these adaptations remain incompletely understood. Here, we combined physiological, biochemical, and transcriptomic analyses to investigate the response of the green microalga *Chlorella* sp. SLA-04 to nitrogen depletion and high-alkalinity growth. Nitrogen depletion redirected carbon toward carbohydrate and lipid storage and was accompanied by coordinated transcriptional changes that suppressed photosynthesis while activating TAG biosynthetic and degradative pathways. The coordinated induction of lipid biosynthetic and degradative pathways is consistent with a model in which TAG synthesis and turnover help maintain redox homeostasis during nutrient stress. In contrast, high-alkalinity growth altered fatty-acid composition and was associated with transcriptional changes involving ion transport, membrane remodeling, and osmotic adaptation. Alkaline growth also altered the expression of mobile genetic elements and genes associated with RNA-mediated genome surveillance, suggesting that adaptation to high pH extends beyond central metabolism. Together, these findings provide a system-level view of how coordinated physiological and transcriptional responses enable *Chlorella* sp. SLA-04 to adapt to nutrient limitations and high-alkalinity growth.

## 1. Introduction

Microalgae inhabit diverse and dynamic aquatic environments in which nutrient availability, pH, and other growth conditions can vary substantially [1,2]. Persistence depends on the ability to coordinate physiological, metabolic, and transcriptional responses that preserve cellular homeostasis despite dynamic environmental challenges. Although many responses to individual environmental stresses have been described, considerably less is known about how concurrent environmental constraints collectively shape microalgal physiology and adaptation [3].

Among the environmental factors that influence microalgal physiology, nutrient availability, specifically nitrogen, is one of the most extensively studied [4]. Nitrogen depletion limits cellular growth while promoting the accumulation of carbon storage compounds such as starch and triacylglycerols (TAGs) [5]. Although photosynthetic activity declines during nitrogen depletion, it does not cease immediately when growth becomes limited, creating a transient imbalance between energy production and the capacity to use that energy for biomass synthesis [6,7]. Microalgae respond by remodeling metabolism, including the accumulation of reduced carbon storage compounds such as starch and TAGs, although the mechanisms by which these metabolic changes contribute to cellular redox homeostasis remain incompletely understood [8,9]. Nitrogen limitation has therefore become a valuable model for investigating stress-induced metabolic reprogramming in microalgae. However, most studies have examined nitrogen or other nutrient deprivation as an individual stress, with only a few investigating the concurrent application of nitrogen limitation and phytochrome stimulation on algal growth [10,11,12]. This provides limited insight into how this response is modified by other environmental conditions encountered in natural and engineered aquatic systems.

High-alkalinity environments impose a distinct set of physiological challenges. High pH alters inorganic carbon and nutrient speciation and challenges pH-dependent transport processes, while the elevated ion concentrations associated with high-alkalinity media can also perturb osmotic balance and membrane homeostasis [13,14]. Maintaining growth under these conditions therefore requires coordinated regulation of ion transport, membrane composition, carbon acquisition, and cellular metabolism. Although some microalgae tolerate sustained growth under highly alkaline conditions, the molecular mechanisms supporting this tolerance remain incompletely understood [15]. Even less is known about how high alkalinity modifies the metabolic response to nitrogen depletion, although these conditions can occur together in natural alkaline environments and are deliberately combined in some engineered cultivation systems [15,16,17].

The green microalga *Chlorella* sp. SLA-04 provides an opportunity to investigate how these distinct environmental constraints shape cellular physiology [18]. *Chlorella* sp. SLA-04 is an oleaginous green alga that was isolated from Soap Lake, a brackish, alkaline lake (100 mM HCO_3_^−^, pH 10) in eastern Washington (USA) [15,18]. SLA-04 tolerates sustained growth under highly alkaline conditions, yet under the conditions tested, it grows faster and produces more biomass at neutral pH, indicating that high-alkalinity growth remains physiologically demanding [15,19]. Here, we combined physiological, biochemical, and transcriptomic analyses to show that nitrogen depletion and high-alkalinity growth elicit distinct but complementary adaptive responses that collectively maintain cellular homeostasis in the alkali-tolerant microalga *Chlorella* sp. SLA-04.

## 2. Materials and Methods

### 2.1. Strain Cultivation Conditions

*Chlorella* sp. SLA-04 was initially cultivated in Bold’s Basal medium (BBM) with an initial pH of 8.7 before being grown in this study in 1.25 L tube photobioreactors with 400 µmol m^−2^s^−1^ light using T5 fluorescent bulbs with a 14:10 light–dark cycle (Supplementary Figure S1) [18]. Cultures received 0.4 L/min of air, and temperature was maintained at 23 °C. Alkaline cultures were grown in BBM supplemented with 100 mM sodium bicarbonate; sodium hydroxide was added to titrate the alkaline medium to pH 10.3 before inoculation. Neutral cultures were grown in BBM and titrated to pH 7 with sodium hydroxide. During cultivation, 5% CO_2_ was mixed in line with the air supply as necessary to buffer the neutral condition if pH rose above 7.2. CO_2_ mixing stopped once pH returned to 7.0.

### 2.2. Dissolved Inorganic Carbon (DIC)

Ten milliliters of culture were harvested and filter-sterilized using a 0.22 µm filter. The supernatant of alkaline conditions was diluted 10 times to bring the inorganic carbon concentration below 100 mg carbon/L. The neutral samples did not need to be diluted. Samples were run on a Formacs-HT TOC analyzer (Skalar, Breda, The Netherlands) to quantify media inorganic carbon concentration. Instrument oxygen flow was set to 200 mL/min, and fresh 4% phosphoric acid (H_3_PO_4_) was supplied every 60 samples. Samples were compared to a standard dilution series composed of molar equivalents of sodium carbonate and sodium bicarbonate.

### 2.3. Media Nitrate Concentration

Szechrome NAS reagent from Polysciences Inc. (Warrington, PA, USA) (Cat# 08762) was prepared by dissolving 0.5 g of the reagent in 100 mL of equal parts concentrated phosphoric (H_3_PO_4_) and sulfuric (H_2_SO_4_) acid [20,21]. The acid mixture was prepared 1 week before adding NAS to minimize background NO_3_^−^ concentrations in the acid. Cultures were harvested (1 mL) and filter-sterilized with a 0.22 µm syringe filter. Samples (20 µL) were transferred to a 96-well plate along with nitrate standards containing 0–50 mg/L N. Acidified NAS (200 µL) was added to each sample, starting with the most concentrated standard. Samples were mixed with a pipet >20 times and left to stand for 30 min. After 30 min, sample absorbance was measured at 570 nm using a Biotek Plate Reader (Aligent, Santa Clara, CA, USA) (Synergy H1), and the concentration was determined using a calibration curve of known standards.

### 2.4. Cell Concentration

Algal cultures (1 mL) were sampled and diluted to approximately 10^6^ cells/mL. Twelve microliters (12 µL) of the diluted samples were added to a hemocytometer with a 10^−4^ mL counting volume, and samples were counted in triplicate. The cell concentration of the original culture was determined using Equation (1).

(1) $$Concentration\left(\frac{Cells}{mL}\right)=\frac{Average\;cell\;count\left(\mathrm{Triplicate}\right)\times \mathrm{Dilution}\;\mathrm{factor}}{10^{-4}\mathrm{mL}}$$

### 2.5. Total Suspended Solids

Algal cultures (10 mL) were collected and filtered through a pre-weighed 0.22 µm filter disk with 25 mm diameter using a vacuum filtration setup, followed by washing the filter 3 times with 5 mL diH_2_O pH 6. Filters containing algal biomass were dried at 80 °C for 24 h and weighed. Total suspended solid (TSS) concentrations (mg/L) were calculated by subtracting the pre-weight of the clean filter from the weight of the dried filter with algal biomass and adjusted for the volume of filtrate used.

### 2.6. Chlorophyll Concentration

Algal cultures (1 mL) were harvested and pelleted in 15 mL Falcon tubes @ 500× *g* for 10 min. The supernatant was discarded, and the pellet was resuspended in 5 mL of 100% methanol. Samples were heated for 5 min at 70 °C, followed by centrifugation for 10 min at 500× *g*. Supernatant was removed and saved. Methanol (5 mL) was added to the pellet, and the extraction was repeated. Then, 1 mL of combined methanol (10 mL total) was added to a cuvette with a 1 cm path length, and absorbance values were collected at 470, 653, and 666 nm using a Genesys 10S UV-VIS spectrophotometer (Thermo Scientific, Waltham, MA, USA). One hundred percent methanol was used as a blank. Chlorophyll A, B, and carotenoid concentrations were calculated using Equation (2) [22].

(2) Chl A (mg/L) = 17.12 × A_666_ − 8.86 × A_653_ Chl B (mg/L) = 32.23 × A_653_ − 14.55 × A_666_ Chl A+B (mg/L) = 2.57 × A_666_ + 23.6 × A_653_ Car (mg/L) = (1000 × A_470_ − 2.86 × (15.65 × A_666_ − 7.34 × A_653_) − 129.2 × (27.05 × A_653_ − 11.21 × A_666_))/221

### 2.7. Nile Red Fluorescence

A total of 40 µL of a Nile Red solution (25 ng/µL dissolved in acetone) was added to each 1 mL of algal culture sample [23,24]. Samples were vortexed, and 200 µL was transferred to a dark-walled 96-well plate and incubated in the dark for exactly 10 min. The microplate was immediately transferred to a Cytation5 microplate reader (Aligent, Santa Clara, CA, USA) with Gen5 software to record fluorescence using an excitation of 480 nm, emission of 580 nm, gain of 75, and read height of 7.0 mm. Relative fluorescence units were recorded and used to compare the approximate lipid content of samples.

### 2.8. Lyophilization of Algal Biomass for FAME and Carbohydrate Analysis

Algal samples (50 mL) were collected from liquid cultures for biomass composition analysis. Samples were pelleted at 3000× *g* for 10 min, the supernatant was removed, and the pellets were frozen at −80 °C. For lyophilization, caps were removed, and tubes with frozen pellets were transferred to a pre-chilled Millrock Technologies freeze dryer set at −40 °C. Samples were lyophilized for 72 h at −40 °C and 100 mTorr. Freeze-dried samples were stored at −20 °C and used for fatty-acid methyl ester (FAME) and carbohydrate analysis.

### 2.9. FAME Analysis

Dried algal biomass (5–15 mg) was transferred to glass vials with Teflon caps. Toluene (0.5 mL) and sodium methoxide (1 mL) were added to each sample. Closed vials were incubated for 30 min at 90 °C and vortexed for 5 s every 10 min. Then, 1 mL of boron trifluoride (14% BF_3_ in methanol) was added, and the incubation step was repeated. Tubes were cooled to room temperature before adding 0.4 mL of hexanes and 0.4 mL of concentrated salt solution (15% NaCl *w*/*w*). The tubes were incubated for 10 min at 90 °C, and the organic layer was separated via centrifugation for 2 min at 500× *g*. The organic layer was transferred to capped GC vials and used for GC-MS analysis of FAME content.

GC–MS analysis was performed as follows. Briefly, using an autosampler, 0.5 μL injections (split 10:1) were performed into a GC-MS (Thermo Fisher Trace 1310 GC coupled to a ISQ7000 mass spectrometer, Thermo Fisher Scientific, Waltham, MA, USA) equipped with an Agilent DB-23 column (60 m long, 0.25 mm diameter, 0.15 µm film thickness). The injector temperature was 240 °C, the transfer line at 230 °C, and the detector (ion source) temperature was 250 °C. The initial column temperature was 50 °C (held for 1 min) and was increased to 175 °C at a rate of 25 °C min^−1^, immediately followed by a ramp at 4 °C min^−1^ to a final temperature of 230 °C, which was held for 10 min before run termination. Helium was used as the carrier gas, and column flow was held constant at 1.0 mL min^−1^ [25]. A 28-component fatty-acid methyl ester standard prepared in methylene chloride (NLEA FAME mixture; Restek, Bellefonte, PA, USA) was used as an external calibration standard for GC-MS retention-time identification and response-curve generation (R^2^ > 0.99). No internal FAME standard was included. FAMEs were identified by retention time, combined with library searches and automated quantification using quantitation and confirmation peaks. Unknowns identified as FAME-like using their fragmentation patterns were quantified using surrogate standard quantification using the response peak with the nearest eluting calibration standard based on retention time. Data analysis was performed using Chromeleon software (Ver. 7.2.10).

### 2.10. Carbohydrate Analysis

Total carbohydrate content reported as glucose equivalents was calculated using a colorimetric anthrone assay [26]. Anthrone reagent was prepared by mixing 10 mg of anthrone and 250 µL of fresh absolute ethanol per reaction. A total of 75% sulfuric acid was added to a final volume of 5 mL per reaction and stirred until the anthrone was dissolved. Dried algal biomass (0.5–1.0 mg) was transferred to glass test tubes with Teflon caps. Samples were re-suspended in 1 mL of water and placed on ice. Glucose standards were prepared from a 1 mg/mL stock. Standards ranged from 0 to 600 µg/mL with a total volume of 1 mL. Five milliliters (5 mL) of ice-cold anthrone reagent were added to each sample and standard. Samples and standards were chilled on ice for 5 min, vortexed, and incubated in boiling water for 10 min. Tubes were placed back on ice for 10 min to cool and vortexed. An aliquot (200 µL) of each sample and standard was transferred to a 96-well plate, and the absorbance was measured at 625 nm. Standards were used to generate a calibration curve, and total carbohydrate content was reported as glucose equivalents.

### 2.11. RNA Extraction and Quantification

Algal samples (50 mL) were collected for RNA sequencing 24 h before and after nitrogen depletion. Samples were spun down (4000× *g* @ 4 °C for 5 min) and flash-frozen in liquid nitrogen to limit RNA degradation. Because neutral and alkaline cultures exhibited different growth rates and depleted nitrogen at different times, RNA-seq sampling was aligned to nitrogen availability rather than culture age. Samples were collected approximately 24 h before and 24 h after nitrogen depletion in each condition (days 2 and 4 for neutral cultures and days 5 and 7 for alkaline cultures, respectively), thereby enabling comparison of analogous physiological stages relative to nitrogen depletion. Total RNA extraction was performed by grinding algal biomass with a mortar and pestle in liquid nitrogen for 5 min. Frozen, ground biomass was transferred to Trizol extraction buffer, and total RNA was purified using the Zymo Research direct-zol RNA miniprep kit (Zymo, Irvine, CA, USA) per manufacturer’s instructions. Purification yielded 7.9–45.5 μg of total RNA per extraction for all 12 samples, with a 260 nm/280 nm ratio ranging from 2 to 2.3 and a 260 nm/230 nm ratio ranging from 2.1 to 2.5. RNA integrity was also confirmed using a 2100 Bioanalyzer (Agilent, Santa Clara, CA, USA). Bioanalyzer samples had distinct 25S and 18S rRNA bands with RNA integrity numbers (RIN) of 7.8–9.5 [27].

### 2.12. RNA Sequencing and Processing

Twelve biological samples were submitted for sequencing (four experimental conditions × three biological replicates). One library from the neutral pre-nitrogen-depletion condition failed during sequencing; therefore, 11 libraries were used for downstream transcriptomic analyses. Total RNA samples were shipped to the Joint Genome Institute (JGI) where mRNA libraries were created, quantified by qPCR, and sequenced using an Illumina platform per the JGI pipeline. Raw reads (.fastq file) were filtered and trimmed using the JGI QC pipeline [28]. Using BBDuk (version 39.01), raw reads were evaluated for artifact sequence by kmer matching (kmer = 25), allowing one mismatch, and detected artifacts were trimmed from the 3′ end of the reads [29,30]. RNA spike-in reads, PhiX reads, and reads containing any Ns were removed. Quality trimming was performed using the phred trimming method set at Q6. Reads under the length threshold, 25 bases or 1/3 of the original read length, whichever was longer, were removed.

Filtered reads from each library were aligned to the reference SLA-04 genome using HISAT2 version 2.2.0 [31]. Additionally, 98.8% of reads mapped back to the SLA-04 genome. Strand-specific coverage bigWig files (fwd and rev) were generated using deepTools v3.1 [32]. FeatureCounts was used to generate the raw gene counts file using gff3 (Version 1.2.1) annotations [33]. Only primary hits assigned to the reverse strand were included in the raw gene counts (-s 2 -p --primary options). Raw gene counts were used to evaluate the level of correlation between biological replicates using Pearson’s correlation and determine which replicates would be used in the differential gene expression (DGE) analysis. Fragments Per Kilobase Million (FPKM) and Transcripts Per Kilobase Million (TPM) normalized gene counts were also generated.

### 2.13. Differential Gene Expression

DESeq2 (version 1.30.0) was used to determine differential gene (DGE) expression between pairs of conditions [34]. The parameters for DGE between conditions were adjusted *p*-value/FDR < 0.05 and log2 fold change > 2. SLA-04 genes were annotated using Mercator 4.5 and mapped to metabolic pathways using KofamKOALA (https://www.genome.jp/tools/kofamkoala/ (accessed on 5 May 2023)) and KEGG (https://www.genome.jp/kegg/ (accessed on 1 May 2023)) mapper [35,36,37]. Volcano plots were generated in RStudio (2023.12.0) using ggplot2 [38].

## 3. Results

### 3.1. Cell Growth

**Figure 1:**
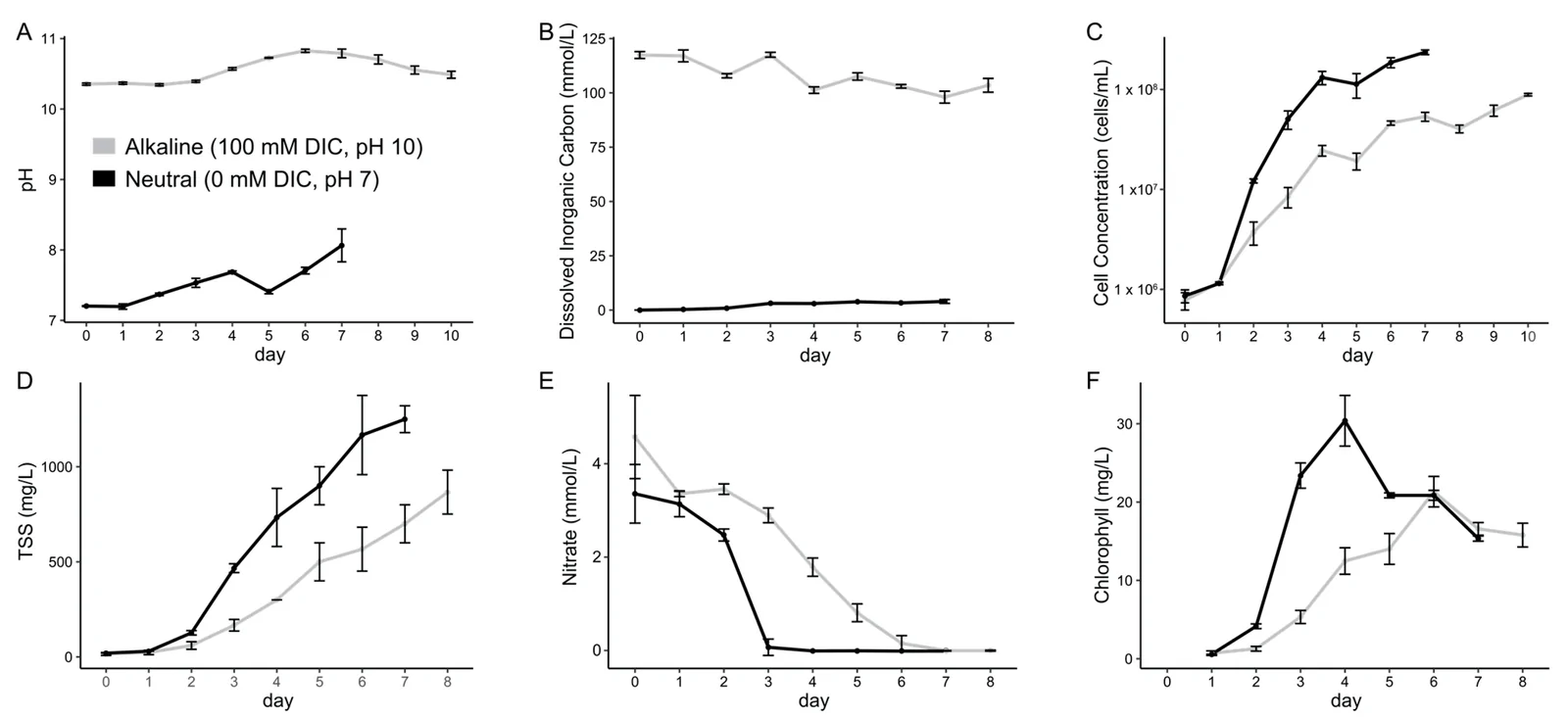
*Chlorella* sp. SLA-04 grows and consumes nitrogen faster in neutral conditions compared to alkaline conditions. SLA-04 was grown in neutral (pH 7, 0 mM added inorganic carbon) and alkaline (pH > 10, 100 mM added inorganic carbon) conditions. (**A**) pH; (**B**) inorganic carbon concentration; (**C**) cell concentration; (**D**) total suspended solids (TSS); (**E**) nitrate concentration; and (**F**) chlorophyll content were monitored and quantified for both conditions. Data represent mean ± SD of n = 3 independent biological replicates. Error bars represent standard deviation.

*Chlorella* sp. SLA-04 was cultivated in BBM at pH 7 without added inorganic carbon (neutral condition) and BBM at pH 10 with 100 mM inorganic carbon (alkaline condition) (Figure 1A,B). The growth rate of SLA-04 was faster in the neutral condition, which was buffered with 5% CO_2_ at 0.4 L/min when the pH exceeded 7.2, resulting in a higher cell concentration and biomass production (Figure 1C,D). Specifically, the doubling time during logarithmic growth was 10.5 h in neutral conditions and 16.5 h in alkaline conditions. Accordingly, the neutral cultures consumed the available nitrogen (nitrate) after approximately 3 days, while the alkaline cultures took approximately 6 days to deplete nitrogen (Figure 1E). Although the neutral condition promoted a greater number of cells and biomass, the higher TSS relative to cell abundance under alkaline conditions suggests greater biomass per cell; however, because cell dimensions were not measured directly, these data do not establish a difference in cell size.

Total chlorophyll concentrations exhibited a more rapid increase in the neutral condition, reaching their peak on day four before decreasing to similar levels as the alkaline condition on day six (Figure 1F). While the total chlorophyll concentrations were higher in the neutral condition, the normalized chlorophyll concentration per cell was, on average, 2.7 times greater in the alkaline condition. Higher TSS relative to cell abundance in alkaline conditions likely contributed but does not fully account for this difference in intracellular chlorophyll concentrations.

### 3.2. Lipid and Carbohydrate Composition

**Figure 2:**
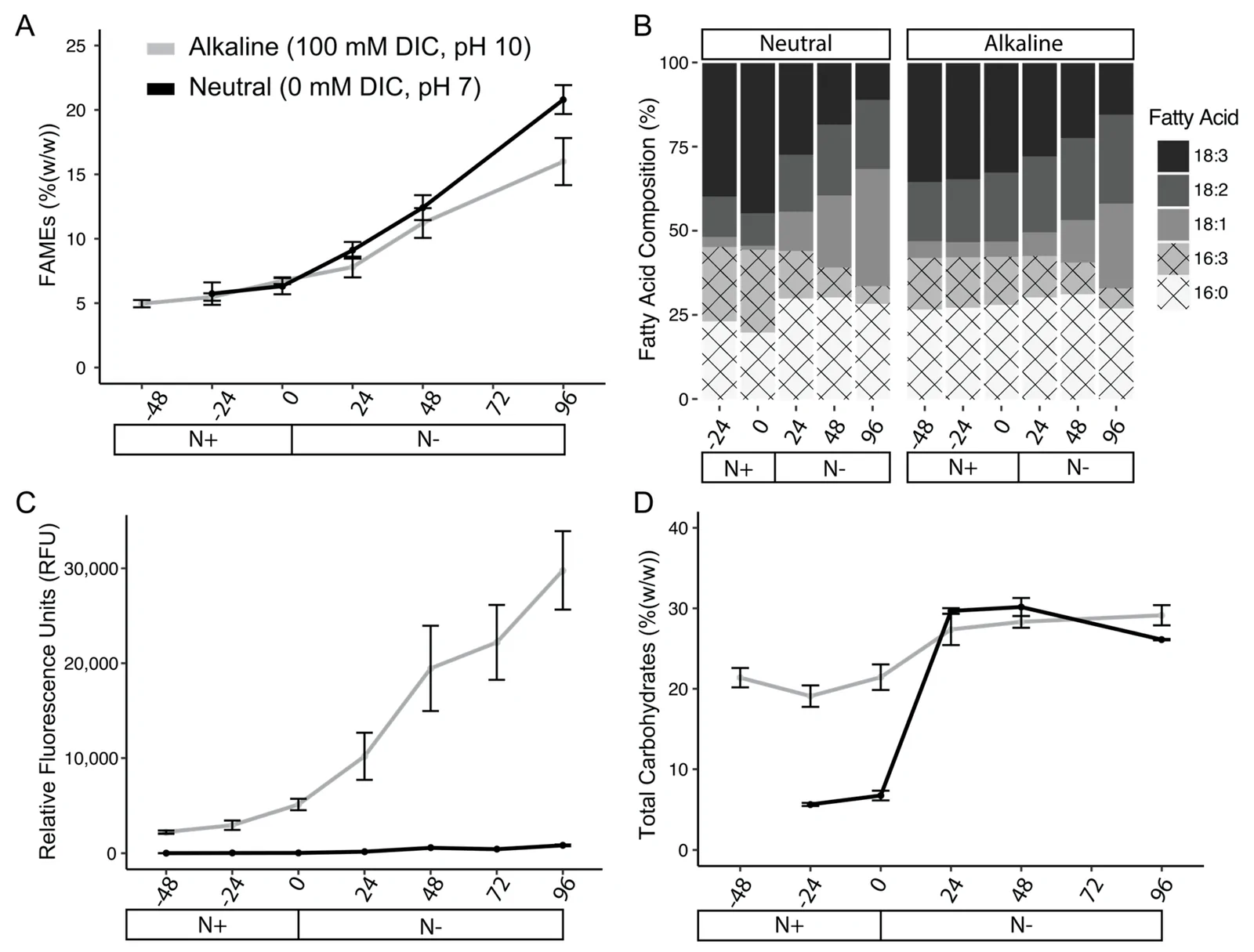
Biomass composition of SLA-04 differs between neutral vs. alkaline conditions and after nitrogen depletion. (**A**) Fatty-acid methyl ester (FAME) content and (**B**) fatty-acid compositions were quantified using GC-MS. The *x*-axis is centered around the point of nitrogen depletion (i.e., day 3 for neutral and day 6 for alkaline conditions) and denotes hours before and after nitrogen depletion. (**C**) Nile Red fluorescence was measured as a rapid fluorescence-based estimate of intracellular hydrophobic material and compared with fatty-acid abundance determined by GC-MS. (**D**) Total carbohydrate content of SLA-04 was also determined. Data represent mean ± SD of n = 3 independent biological replicates. Error bars represent standard deviation.

Cellular fatty acids were converted to fatty-acid methyl esters and quantified by GC-MS to determine total fatty-acid abundance and composition. In both alkaline and neutral conditions, fatty-acid abundance increased following nitrogen depletion (Figure 2A). After centering the data around nitrogen depletion (neutral = day 3; alkaline = day 6), cells cultivated in neutral and alkaline conditions had nearly identical fatty-acid accumulation profiles prior to nitrogen depletion (Figure 2A). At nitrogen depletion, neutral and alkaline cultures contained 6.3% and 6.7% total lipids weight/weight and increased to 12.4% and 11.2%, respectively, 48 h after nitrogen depletion (Supplementary Table S1). A significant difference in fatty-acid abundance was observed only four days (96 h) after nitrogen depletion, with lower levels of total fatty acids in the alkaline condition (Figure 2A, Supplementary Table S1).

Five major fatty-acid species were detected in the transesterified samples as FAMEs in both neutral and alkaline conditions. These fatty acids ranged from C16–C18 in length and contained 0–3 degrees of unsaturation (Figure 2B). The overall fatty-acid length within SLA-04 was impacted by nitrogen availability. Before nitrogen depletion, fatty acids within the neutral and alkaline conditions were composed of 54.7% and 57.7% C18 fatty acids, respectively, with the remaining fraction being C16 fatty acids (Supplementary Table S1). Four days after nitrogen depletion, both cultures increased in C18 fatty-acid composition to 66.3% and 67.0% of total fatty acids (Figure 2B, Supplementary Table S1). In addition to increasing chain length after nitrogen depletion, fatty acids in both conditions became more saturated. After nitrogen depletion, both neutral and alkaline cultures showed a significant decrease in C18:3 (*p*-value of 8.68 × 10^−5^ and 0.0147, respectively) and C16:3 (*p*-value of 0.000215 and 0.00569, respectively) fatty acids and an increase in C18:1 fatty acid methyl esters (FAMEs) (*p*-value of 0.000276 and 0.0261, respectively) compared to log phase cultures (Supplementary Table S2).

While SLA-04 grown in neutral and alkaline conditions showed similar trends of increased saturation and length after nitrogen depletion, fatty-acid composition between conditions was statistically different based on a two-way ANOVA analysis. During log phase growth, SLA-04 grown in neutral conditions contained significantly more unsaturated fatty acids, with statistically more C18:3 (*p*-value of 0.0039) and C16:3 (*p*-value of 0.0003) compared to the alkaline condition (Supplementary Table S1). After nitrogen depletion, this trend was reversed, and fatty acids from SLA-04 grown in alkaline conditions were significantly more unsaturated. After nitrogen depletion, SLA-04 grown in alkaline conditions had significantly more C18:3 (*p*-value of 0.0190) and C18:2 (*p*-value of 0.0003), while neutral conditions resulted in more C18:1 (*p*-value of 0.0317).

Nile Red fluorescence was substantially higher in alkaline-grown cultures and increased markedly following nitrogen depletion, whereas fluorescence remained comparatively low in neutral cultures (Figure 2C). However, Nile Red fluorescence did not consistently correspond with total fatty-acid abundance measured by GC-MS, indicating that Nile Red fluorescence was not a reliable proxy for total fatty-acid content under the conditions tested here (Figure 2A) [23,24,39,40]. The discrepancy suggests that physiological changes associated with alkaline growth influence Nile Red fluorescence independently of total fatty-acid abundance. Because Nile Red partitions into hydrophobic cellular environments, differences in the abundance, composition, or accessibility of intracellular constituents may contribute to the fluorescence signal. Alkaline-grown cells also contained substantially more carbohydrates before nitrogen depletion, but carbohydrate concentrations later converged while the difference in Nile Red fluorescence persisted (Figure 2D), indicating that carbohydrate accumulation alone cannot explain the fluorescence pattern. These findings demonstrate that Nile Red fluorescence does not provide a reliable quantitative estimate of fatty-acid abundance across the alkaline condition tested here and emphasize the importance of validation using direct biochemical measurements [40].

### 3.3. Photosynthesis

**Figure 3:**
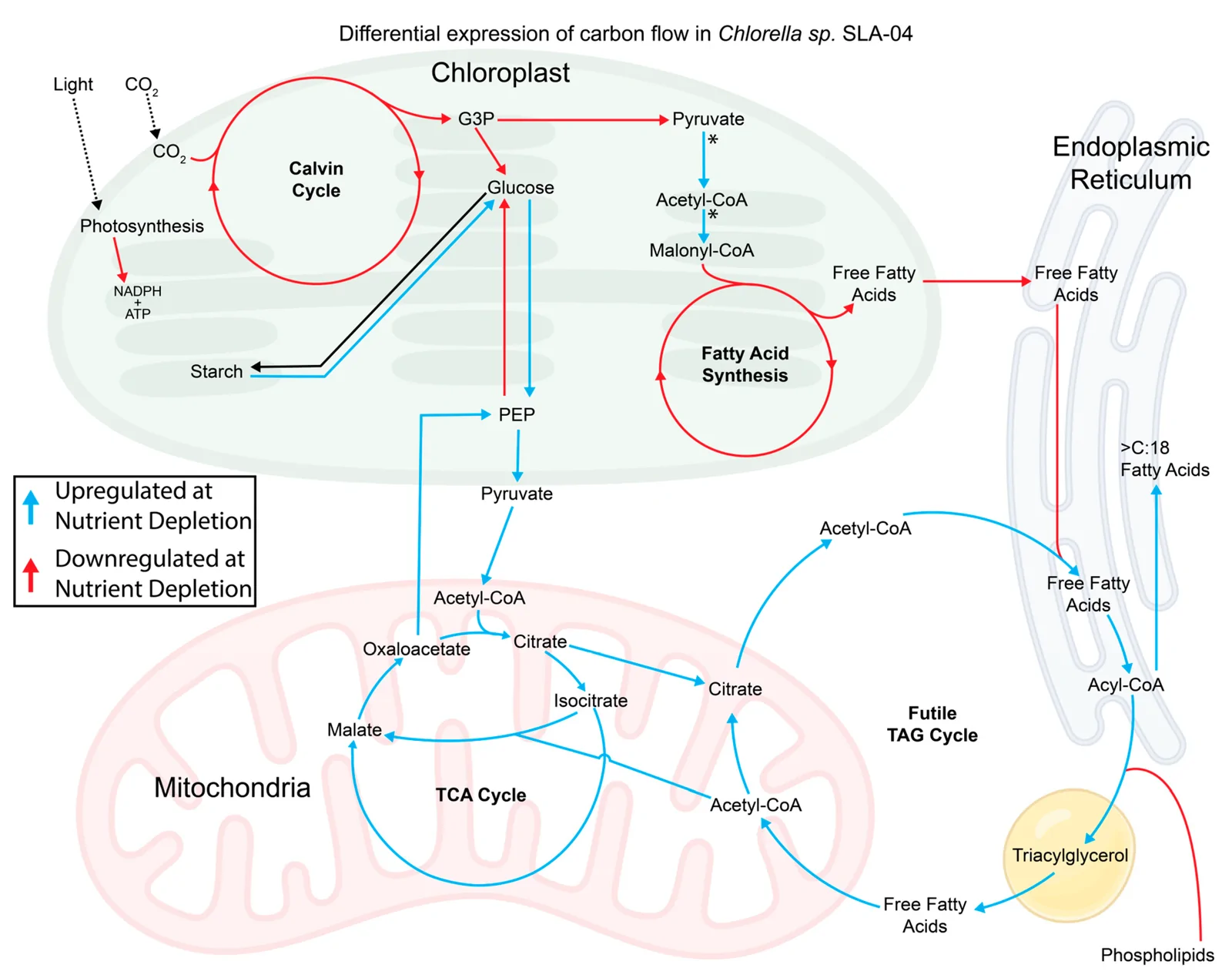
The expression of genes involved in carbon metabolism is altered after nitrogen depletion. Blue arrows indicate upregulated pathways in nitrogen-deplete conditions. Red arrows indicate downregulated pathways. Black arrows indicate pathways with no differential expression. * genes upregulated in alkaline conditions (with and without nitrogen). See Supplementary Figure S4 for an expanded version of carbon metabolism gene expression.

Total RNA was extracted in triplicate from *Chlorella* sp. SLA-04 cultures 24 h before and after nitrogen depletion from both neutral and alkaline conditions (Supplementary Figure S2). Enriched mRNA was deep-sequenced using an Illumina platform. Transcripts were mapped back to annotated genes within the SLA-04 genome using HISAT2, and FeatureCounts was used to calculate expression levels of individual genes. Differential gene expression profiles between conditions were generated using DESeq2. Principal component analysis of samples showed that biological replicates of each condition formed distinct clusters (Supplementary Figure S3). Differential gene expression was compared between neutral and alkaline cultures while also comparing nitrogen-replete versus nitrogen-deplete conditions. To identify mechanisms of fatty-acid and carbohydrate accumulation, we explored how genes involved in central carbon metabolism were differentially expressed between conditions (Figure 3). The highest levels of differential expression within carbon metabolism were observed when comparing pre- and post-nitrogen depletion time points for each condition (Supplementary Figure S3).

Expression levels for most genes involved in photosynthesis decreased after nitrogen limitation in both neutral and alkaline conditions (Figure 3). Downregulation was observed in genes coding for photosystem II (PSII), photosystem I (PSI), and ATP synthase proteins (Supplementary Figure S3). Genes involved in carbon fixation were also downregulated after nitrogen depletion. Terpenoid biosynthesis was downregulated in nitrogen-limited conditions, suggesting that photosynthetic accessory pigment production was also reduced. Select photosynthetic genes, including SLA-04_08060-RA, SLA-04_02138-RA, and SLA-04_00479-RA, involved in photo-oxidation repair and synthesizing chlorophyll showed opposite expression profiles with significant upregulation after nitrogen depletion (Supplementary Table S3).

### 3.4. Starch and Sugar Metabolism

Genes involved in starch elongation were slightly upregulated (starch synthase, SLA-04_06947-RA, and starch branching enzyme, SLA-04_05722-RA) during nitrogen-limited conditions. However, ADP-glucose pyrophosphorylase SLA-04_05039-RA, which produces ADP-glucose, the first committed step of starch synthesis, was downregulated (Supplementary Figure S4). A clearer trend of upregulation was observed in starch degradation genes (Figure 3). Genes involved in starch degradation had their lowest levels of expression in neutral media replete with nitrogen. In contrast, these genes were upregulated in all alkaline conditions and neutral nitrogen-deplete environments. Certain genes involved in starch degradation (i.e., amylase, SLA-04_02883-RA) showed strong upregulation in alkaline conditions only, while expression levels were low in neutral conditions, even after nitrogen depletion (Supplementary Tables S3 and S4).

Genes related to glucose catabolism were upregulated after nitrogen depletion in neutral and alkaline conditions (Figure 3, Supplementary Figure S4). Upregulation was observed in all irreversible steps of glycolysis and the oxidation of pyruvate to acetyl-CoA. While catabolic reactions were upregulated, gluconeogenesis was downregulated after nitrogen depletion. Together, the upregulation of catabolic pathways like starch degradation, glycolysis, and pyruvate oxidation with the downregulation of gluconeogenesis suggests that nitrogen depletion may trigger gene expression changes in SLA-04 that modify metabolism for the breakdown of starches and facilitate the production of other macromolecules like storage lipids.

### 3.5. Fatty-Acid Synthesis

Gene expression related to fatty-acid synthesis in the chloroplast was downregulated during nitrogen depletion (Figure 3, Supplementary Figure S4). Almost all fatty-acid synthesis genes had the highest levels of expression in neutral nitrogen-replete conditions. Levels of expression were slightly lower in alkaline nitrogen-replete conditions, but the most significant downregulation occurred once both neutral and alkaline cultures became nitrogen-deplete. While genes involved in fatty-acid synthesis were downregulated in the chloroplast after nitrogen depletion, genes involved in the production of fatty-acid precursors had different expression trends. Fatty-acid synthesis requires acetyl-CoA and malonyl-CoA to elongate fatty acids. Genes involved in the production of acetyl-CoA from pyruvate and the conversion of acetyl-CoA to malonyl-CoA within the chloroplast were upregulated in both alkaline conditions (pre- and post-nitrogen depletion) and downregulated in both pre- and post-nitrogen depletion in neutral conditions (Supplementary Figure S4).

### 3.6. Lipid Synthesis

While genes related to fatty-acid metabolism in the chloroplast were downregulated after nitrogen depletion, carbon metabolism in other cellular compartments was significantly upregulated. Genes related to the synthesis, elongation, and degradation of triacylglycerol (TAG) storage lipids were upregulated in nitrogen-deplete conditions (Figure 3, Supplementary Figure S4). TAG biosynthesis and degradation were also upregulated in nitrogen-replete alkaline conditions, suggesting that both nitrogen availability and alkalinity impact the expression of genes related to fatty-acid synthesis (Supplementary Figure S4). In contrast to TAG synthesis, elongation, and degradation, genes related to the conversion of phospholipids to TAGs were downregulated in nitrogen-deplete conditions.

### 3.7. Fatty-Acid Catabolism and Mitochondrial Carbon Metabolism

TAGs can be stored in lipid bodies or catabolized to release fatty acids. Fatty acids produced by TAG catabolism can be converted to acetyl-CoA through β-oxidation, which occurs primarily in peroxisomes in green algae [41]. In SLA-04, genes associated with β-oxidation were upregulated after nitrogen depletion (Figure 3, Supplementary Figure S4). Acetyl-CoA or other products generated through fatty-acid catabolism may subsequently support mitochondrial TCA-cycle metabolism or the glyoxylate shunt. Both the glyoxylate shunt and TCA-cycle pathways were significantly upregulated under neutral and alkaline conditions after nitrogen depletion.

In addition to the upregulation of central TCA genes, we also saw an upregulation of genes capable of converting TCA cycle intermediates to cytosolic acetyl-CoA. ATP citrate lyase (ACLY) was upregulated in alkaline nitrogen-replete and both nitrogen-deplete conditions compared to SLA-04 from nitrogen-replete neutral conditions (Figure 3, Supplementary Figure S4). ACLY is a key enzyme for replenishing cytosolic acetyl-CoA for TAG synthesis and elongation. The upregulation of TAG synthesis, TAG degradation, beta-oxidation, and ACLY in nitrogen-deplete conditions suggests that stressful conditions trigger SLA-04 to shift stored carbon between lipids and the TCA cycle.

### 3.8. Genes Involved in Adaptation to Nitrogen Depletion

**Figure 4:**
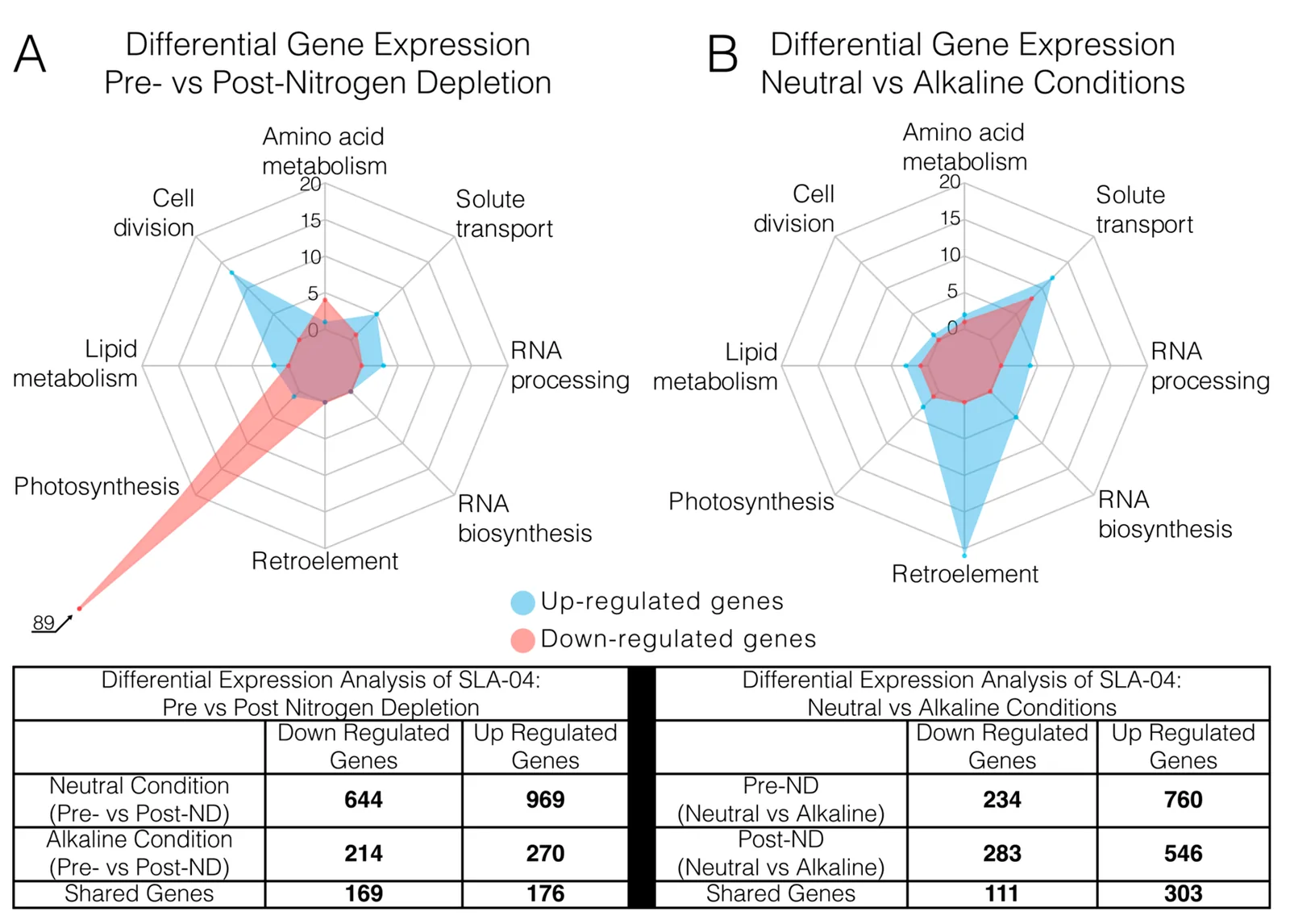
Media nitrogen availability and alkalinity impact gene expression in SLA-04 differently. (**A**) Gene clusters based on function with the greatest up- or downregulation in nitrogen-replete vs. nitrogen-deplete conditions. Red represents downregulated gene clusters, and blue represents upregulated gene clusters. Total gene clusters with differential expression between nitrogen-replete and -deplete conditions are noted in the table below the figure. (**B**) Gene clusters based on function with the greatest up- or downregulation in alkaline vs. neutral conditions. Total gene clusters with differential expressions between alkaline and neutral conditions are noted in the table below the figure.

In addition to specifically looking at differential expressions within carbon metabolism, we also identified genes with the highest overall differential gene expression. To identify genes that correlate with nitrogen availability, we analyzed neutral (Pre-ND neutral vs. Post-ND neutral) and alkaline (Pre-ND alkaline vs. Post-ND alkaline) conditions independently to minimize the potential influence of alkalinity on gene expression. After nitrogen depletion in the neutral condition, 644 genes were significantly downregulated while 969 were upregulated (Supplementary Figure S5a). After nitrogen depletion in the alkaline conditions, 214 genes were downregulated, while 270 were upregulated (Supplementary Figure S5b). Together, 169 downregulated and 176 upregulated genes were shared between neutral and alkaline conditions after nitrogen depletion (Figure 4A). Of the shared genes, 126 downregulated genes and 97 upregulated genes have predicted functions. We hypothesize that shared genes between the neutral and alkaline conditions play critical roles in adaptation to nitrogen-deplete conditions.

Shared genes that were differentially expressed after nitrogen depletion in neutral and alkaline conditions clustered into multiple metabolic pathways. Of the 126 functionally annotated genes that were downregulated, 89 were involved in photosynthesis (Figure 4A, Supplementary Table S3). The downregulation of photosynthetic genes was complemented by significant upregulation of oxidative stress and photosynthetic repair genes. Genes that synthesize antioxidants, including carotenoids and serotonin, were upregulated (Supplementary Table S3). Additionally, superoxide dismutase genes were highly upregulated in nitrogen-deplete conditions. This suggests a strong global downregulation of photosynthesis and increased photo-repair and oxidative stress protection in nitrogen-deplete conditions.

The second highest downregulation was observed in genes involved in amino acid synthesis and translation. In nitrogen-deplete conditions, where nitrogen levels are insufficient for high levels of protein synthesis [42,43], specific genes involved in nitrogen scavenging were upregulated. Significant upregulation was also seen in genes involved in the transport of nitrogen-rich compounds, including amino acids, uric acid, and nucleotides (Figure 4A, Supplementary Table S3). Additionally, catabolic ammonium generation through deaminase activity was significantly upregulated. Transporting nitrogen-rich molecules and recycling amines may be used to supplement the low nitrogen availability in the stationary phase [44].

This analysis also identified genes involved in fatty-acid biosynthesis and TAG accumulation. Diacylglycerol acyltransferase (DGAT) was highly upregulated in nitrogen-deplete conditions (Supplementary Table S3). This gene has previously been described as being involved in lipid accumulation under nitrogen-deplete conditions and serves as evidence that our analysis is identifying genes critical for lipid acclimation to low-nutrient conditions [45,46]. In addition to DGAT, we also measured an up-regulation of a caleosin-type peroxygenase. Caleosin-type peroxygenase enzymes are involved in abiotic stress responses and lipid droplet storage in plants [47]. The increased expression may suggest that peroxygenase activity plays a role in TAG accumulation and processing in SLA-04 after nitrogen depletion, or simply in regulating and controlling oxidative stress.

### 3.9. Genes Involved in Adaptation to Alkaline Conditions

To identify genes associated with alkaline growth, we analyzed differential gene expressions between neutral and alkaline conditions. Samples harvested 24 h before and 24 h after nitrogen depletion were compared independently to minimize the effects of nitrogen depletion on gene expression (neutral Pre-ND vs. alkaline Pre-ND and neutral Post-ND vs. alkaline Post-ND). When comparing the neutral versus alkaline conditions prior to nitrogen depletion, 234 genes were identified as significantly downregulated while 760 were upregulated (Supplementary Figure S6a). Comparing the alkaline versus neutral condition after nitrogen depletion showed that 283 genes were significantly downregulated and 546 were upregulated (Supplementary Figure S6b). A total of 111 downregulated and 303 upregulated genes were shared between both analyses (Figure 4B). Of the shared genes, 47 downregulated and 149 upregulated genes have predicted functions. Shared genes between these two comparisons are expected to play a role in adaptation to alkaline growth.

Interestingly, retroelements were the most abundant group of genes significantly upregulated in alkaline conditions. The SLA-04 genome contains 26 Copia-like retrotransposons integrated throughout the 13 nuclear chromosomes [18]. Of these 26 retrotransposons, 21 are highly upregulated in alkaline conditions (Figure 4B, Supplementary Table S4). Retroelements are commonly activated in stressful conditions [48,49,50]. Alkaline-induced stress is supported by the upregulation of superoxide dismutase and desiccation response proteins (Supplementary Table S4). In addition to retroelements, we also saw an upregulation in RNA processing and biosynthesis genes. Specifically, we saw an upregulation of Dicer and two T2 RNases. Dicer is an RNase III family enzyme responsible for processing double-stranded RNA (dsRNA) into small interfering RNAs (siRNAs) that provide sequence-specific post-transcription gene silencing [51,52]. The coordinated upregulation of retrotransposons and RNA-processing or RNA-silencing-associated genes is consistent with stress-induced activation of mobile genetic elements accompanied by cellular mechanisms that may limit their expression or propagation.

Solute transport was also modified during alkaline growth. Multiple potassium, sodium, sulfate, calcium, and phosphate transporters were highly upregulated in SLA-04 during growth in alkaline conditions (Figure 4B, Supplementary Table S4). In contrast, iron accumulation and iron oxidation genes were downregulated. SLA-04 may be adapting to the higher osmotic stresses of alkaline conditions by utilizing alternative transport mechanisms to acquire necessary nutrients and maintain intracellular homeostasis. Alkaline growth also resulted in opposing transcriptional responses among ammonium transporters, with AMT1-1 downregulated and AMT1-2 strongly upregulated (Supplementary Table S4) [53]. Transcriptional profiles of ammonium transporters are highly diverse, but differential expressions in response to nutrient and light stress are also observed in plants [54,55]. Because nitrate is the nitrogen source supplied in BBM, these changes are unlikely to reflect a simple response to extracellular ammonium availability and may instead reflect altered intracellular ammonium recycling or redistribution under alkaline conditions. The physiological significance of these opposing responses remains uncertain.

## 4. Discussion

Nitrogen deprivation is a well-established trigger of metabolic reprogramming in microalgae, and transcriptomic studies across diverse species have revealed coordinated changes in photosynthesis, nitrogen acquisition, central carbon metabolism, and lipid storage [11,12,56,57,58]. These physiological responses to nutrient availability have important consequences for microalgal cultivation and bioprocessing performance [58,59]. In contrast, far less is known about how these responses are modified by growth under high-alkalinity conditions. Our results are consistent with these general features of the nitrogen-stress response but extend this work by directly comparing nitrogen depletion with a distinct environmental stress in the same organism. This comparison reveals that SLA-04 does not respond to these conditions through a common generalized stress program. Instead, nitrogen depletion primarily redirects carbon allocation toward carbohydrate and lipid storage, whereas alkaline growth produces a distinct response characterized by changes in fatty-acid composition and transcriptional remodeling of pathways associated with membrane and cellular homeostasis. By integrating transcriptomic, biochemical, and physiological measurements, these results distinguish a conserved nitrogen-depletion response from the physiological program associated with alkaline growth in SLA-04. This study confirms that stress adaptation in *Chlorella* sp. SLA-04 is achieved through the coordinated implementation of stress-specific physiological programs rather than a generalized stress response.

Nitrogen depletion consistently redirects carbon away from biomass production and toward the accumulation of starch and TAGs in diverse microalgal species [4,8,57,60]. Our physiological measurements and transcriptomic analyses are consistent with this well-established response, demonstrating increased carbon storage accompanied by reduced expression of photosynthesis-associated genes and coordinated remodeling of lipid metabolism (Figure 3 and Figure 4). However, the simultaneous induction of both lipid biosynthetic and degradative pathways suggests that TAG metabolism may serve functions beyond long-term carbon storage. During nitrogen limitation, cellular growth becomes constrained while photosynthetic electron transport can continue, creating a transient imbalance between the production and utilization of reducing equivalents. We propose that the coordinated induction of anabolic and catabolic lipid pathways is consistent with a model in which dynamic TAG turnover helps dissipate excess reducing power while maintaining carbon reserves during nutrient stress. Although direct measurements of metabolic flux will be required to test this hypothesis, our findings provide transcriptomic evidence that TAG metabolism may contribute to cellular redox homeostasis in addition to carbon storage.

In contrast to nitrogen depletion, high-alkalinity growth elicited a distinct adaptive program centered on maintaining membrane integrity and cellular homeostasis. Elevated pH and alkalinity alter inorganic carbon chemistry, ion gradients, and osmotic conditions, requiring coordinated physiological adjustments to sustain growth [61,62]. Consistent with these challenges, SLA-04 exhibited broad transcriptional remodeling of ion transport systems and accumulated substantially more carbohydrates before nitrogen depletion than cells grown under neutral conditions (Figure 2). Although the composition and subcellular distribution of these carbohydrates were not determined, in addition to serving as an electron sink, non-structural sugars can act as compatible solutes and maintain intracellular homeostasis during extreme osmotic stress [63,64,65]. The observed changes in sodium, potassium, sulfate, calcium, phosphate, and ammonium transporters further suggest that alkaline-grown cells modify solute transport to maintain intracellular pH, nutrient acquisition, and ionic balance. Together, these responses indicate that adaptation to high alkalinity extends beyond central carbon metabolism and requires coordinated regulation of membrane-associated and homeostatic processes.

Following nitrogen depletion, cells grown under alkaline conditions also retained a more unsaturated fatty-acid profile than cells grown under neutral conditions. Because lipid unsaturation can help preserve membrane fluidity and the function of membrane-associated protein complexes under osmotic and physicochemical stress, this pattern is consistent with membrane remodeling as an important component of alkaline adaptation [66,67,68]. While FAME measurements reflect total cellular fatty acids and do not establish that the altered lipids are specifically incorporated into cellular membranes, greater lipid unsaturation can also increase susceptibility to oxidation [69,70,71]. The persistence of a more unsaturated fatty-acid profile under alkaline conditions therefore suggests a potential physiological tradeoff in which lipid remodeling that supports membrane function may increase vulnerability to oxidative damage. Direct measurements of membrane fluidity, lipid localization, and lipid peroxidation will be required to test this interpretation.

These observations demonstrate that nitrogen depletion and high alkalinity do not simply activate a common stress program. Instead, they impose distinct physiological demands that can produce overlapping responses with different potential functions, as well as adaptations that may introduce competing physiological costs. Carbohydrate accumulation, for example, is associated with both conditions but may contribute primarily to carbon management during nitrogen depletion and to osmotic adjustment during alkaline growth. Similarly, increased fatty-acid unsaturation may support membrane function under alkaline conditions while increasing susceptibility to oxidative damage. The response of SLA-04 therefore reflects the integration of stress-specific physiological demands rather than the uniform deployment of a generalized stress response.

These coordinated responses reflect the fundamentally different physiological constraints imposed by nutrient limitation and alkaline growth. In addition to these metabolic adaptations, alkaline growth was associated with increased expression of mobile genetic elements and genes involved in RNA-mediated genome surveillance. Although we did not directly measure transposon activity, these coordinated transcriptional changes are consistent with stress-induced activation of mobile genetic elements accompanied by cellular responses that may help preserve genome integrity.

Microalgae in natural and engineered environments rarely encounter environmental stresses in isolation, yet adaptive responses are commonly investigated one at a time. By integrating physiological, biochemical, and transcriptomic analyses across nutrient-replete and nitrogen-deplete cultures grown under neutral and high-alkalinity conditions, this study provides a system-level framework for understanding how distinct environmental constraints shape cellular physiology. Our findings demonstrate that adaptation to nutrient limitation and alkaline growth involves coordinated, but fundamentally different, physiological programs that collectively preserve cellular homeostasis. More broadly, the results illustrate how apparently similar phenotypes can arise under different environmental conditions while serving distinct physiological functions, and how adaptation to one constraint may impose costs under another. These results advance our understanding of stress adaptation in alkali-tolerant microalgae and provide a framework for interpreting how photosynthetic microorganisms integrate concurrent environmental demands.

## Data Availability

The sequencing data generated in this study have been deposited in the NCBI BioProject database under accession number PRJNA766673.

## Supplementary Materials

The following supporting information can be downloaded at: https://www.mdpi.com/article/10.3390/microorganisms14092092/s1, Supplementary Figure S1: The modified aquarium used for algal growth experiments. Supplementary Table S1: Fatty acid composition of SLA-04 grown in neutral and alkaline conditions. Supplementary Table S2: Statistical analysis of Fatty acid composition of SLA-04 Before and after Nitrogen Depletion. Supplementary Figure S2: Sampling timepoints and sequencing coverage of this experiment. Supplementary Figure S3: Principal Component Analysis (PCA) analysis of RNA-sequencing data. Supplementary Figure S4: Expanded version of Figure 3 in the manuscript providing more detailed differential expression of genes involved in carbon flow between growth conditions. Supplementary Figure S5: Volcano plots showing differential gene expression (DGE) of SLA-04 pre vs post-nitrogen depletion. Supplementary Figure S6: Volcano plots showing differential gene expression (DGE) of SLA-04 grown in neutral vs alkaline media. Supplementary Table S3: Table of genes with statistically significant differential genes expression between pre- versus post-nitrogen depletion. Supplementary Table S4: Table of genes with statistically significant differential genes expression between neutral versus alkaline cultivation conditions.
