# Supplementary material for: Stress-Induced Metabolic Reprogramming in the Green Microalga Chlorella sp. SLA-04
Source: Microorganisms. 2026 Sep 18;14(9):2092. doi: 10.3390/microorganisms14092092 (PMC13609647; doi:10.3390/microorganisms14092092)
Supplement: Supplementary file 1 [file microorganisms-14-02092-s001.zip › microorganisms-4501337-supplementary.pdf]

Supplementary data

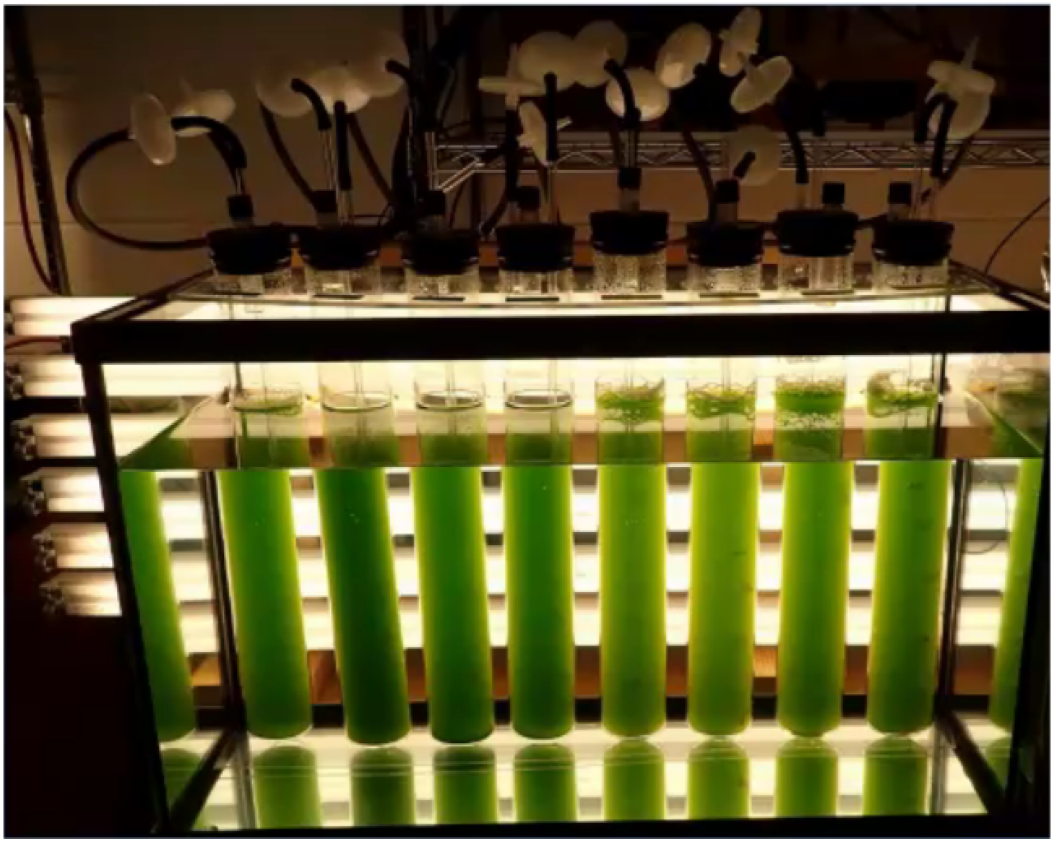

Supplementary Figure S1: The modified aquarium used for algal growth experiments. The photo shows eight cultures of SLA-04 in ~1.4 L culture tubes suspended in an aquarium filled with water. Each growth tube was initially filled with 1.25 L media and capped with black butyl rubber stoppers. Ports were present in each stopper for delivery of filtered gases through a glass tube reaching the bottom of the reactors and venting as well as for sampling. The photobioreactors were illuminated using T5 fluorescent bulbs at a light intensity of 400 µmol $m^{-2}s^{-1}$ using a 14:10 light-dark cycle. Cultures received 0.4 L/min of air (plus 5% CO_2 for the neutral (low alkalinity) conditions when the pH exceeded 7.2). The temperature was maintained at 23ºC using a recirculation chiller.

Supplementary Table S1: Fatty acid composition of SLA-04 grown in neutral and alkaline conditions. ND represents the time of nitrogen depletion (approximately 3 days for the neutral condition and approximately 6 days for the alkaline conditions) and days before(-) or after(+) ND. Bolded values represent data significantly different between the alkaline and neutral conditions of the same timepoint. P-values were generated using R-studio t.test command.

Percent Biomass as Fatty acids (w/w)					
Time	Neutral	Neutral STDEV	Alkaline	Alk STDEV	Pvalue
ND-2	NA	NA	4.95%	0.29%	NA
ND-1	5.74%	0.88%	5.47%	0.29%	0.6613
ND	6.32%	0.63%	6.73%	0.27%	0.3765
ND+1	8.75%	0.41%	7.81%	0.81%	0.1726
ND+2	12.42%	0.97%	11.23%	1.15%	0.2429
ND+4	**20.81%**	**1.13%**	**16.00%**	**1.82%**	**0.0358**

Percent Fatty Acid as 18:3 (w/w)					
Time	Neutral	Neutral STDEV	Alkaline	Alk STDEV	Pvalue
ND-2	NA	NA	35.31%	1.40%	NA
ND-1	**39.66%**	**0.21%**	**34.52%**	**0.73%**	**0.0039**
ND	**44.59%**	**0.78%**	**32.49%**	**0.36%**	**0.0004**
ND+1	27.22%	0.42%	27.72%	1.64%	0.6568
ND+2	18.27%	0.64%	22.24%	2.07%	0.0687
ND+4	**10.92%**	**0.36%**	**15.31%**	**1.28%**	**0.0190**

Percent Fatty Acid as 16:3 (w/w)					
Time	Neutral	Neutral STDEV	Alkaline	Alk STDEV	Pvalue
ND-2	NA	NA	15.32%	0.70%	NA
ND-1	**22.08%**	**0.45%**	**14.97%**	**0.17%**	**0.0003**
ND	**24.58%**	**0.07%**	**14.31%**	**0.47%**	**0.0004**
ND+1	**14.13%**	**0.32%**	**12.32%**	**1.06%**	**0.0017**
ND+2	8.91%	0.77%	9.40%	0.76%	0.4720
ND+4	5.26%	0.48%	5.99%	0.43%	0.2225

Percent Fatty Acid as 18:2 (w/w)					
Time	Neutral	Neutral STDEV	Alkaline	Alk STDEV	Pvalue
ND-2	NA	NA	17.61%	0.31%	NA
ND-1	**12.00%**	**0.36%**	**18.68%**	**0.39%**	**2.89E-05**
ND	**9.67%**	**0.12%**	**20.50%**	**0.38%**	**0.0004**
ND+1	**16.94%**	**0.34%**	**22.59%**	**0.44%**	**0.0001**
ND+2	**21.08%**	**0.19%**	**24.38%**	**0.50%**	**0.0034**
ND+4	**20.54%**	**0.20%**	**26.44%**	**0.21%**	**0.0003**

Percent Fatty Acid as 16:0 (w/w)					
Time	Neutral	Neutral STDEV	Alkaline	Alk STDEV	Pvalue
ND-2	NA	NA	26.78%	1.58%	NA
ND-1	**23.25%**	**0.87%**	**27.30%**	**0.26%**	**0.0100**
ND	**19.93%**	**0.63%**	**28.14%**	**0.30%**	**0.0004**
ND+1	30.05%	0.62%	30.36%	0.69%	0.6051
ND+2	30.33%	0.34%	31.30%	0.96%	0.2164
ND+4	28.46%	0.26%	27.08%	1.21%	0.1806

Percent Fatty Acid as 18:1 (w/w)					
Time	Neutral	Neutral STDEV	Alkaline	Alk STDEV	Pvalue
ND-2	NA	NA	4.98%	0.68%	NA
**ND-1**	**3.00%**	**0.33%**	**4.53%**	**0.43%**	**0.00962**
**ND**	**1.23%**	**0.13%**	**4.56%**	**0.18%**	**2.96E-05**
**ND+1**	**11.65%**	**0.39%**	**7.02%**	**1.60%**	**0.0317**
**ND+2**	**21.41%**	**1.26%**	**12.67%**	**3.31%**	**0.0320**
**ND+4**	**34.82%**	**0.38%**	**25.18%**	**3.02%**	**0.0288**

Supplementary Table S2: Statistical analysis of Fatty acid composition of SLA-04 Before and after Nitrogen Depletion. ND represents the time of nitrogen depletion (approximately 3 days for the neutral condition and approximately 6 days for the alkaline conditions). Fatty acid composition of alkaline and neutral samples prior to nitrogen depletion was compared to composition 1 or 2 days after ND. Bolded values represent p-values generated using R-studio t.test command.

Alkaline Condition	C18:3 Fatty Acid Content		C16:3 Fatty Acid Content		C18:1 Fatty Acid Content	
**t-Test: Paired**	*ND*	*ND+1*	*ND*	*ND+2*	*ND*	*ND+2*
Mean	0.32489456	0.27719092	0.14307484	0.09403126	0.04564951	0.12672856
Variance	1.3027E-05	0.00026792	2.2533E-05	5.7767E-05	3.3103E-06	0.00109549
Observations	3	3	3	3	3	3
Pearson Correlation	0.60291104		-0.0442549		-0.1368256	
Df	2		2		2	
t Stat	5.70564612		9.2964904		-4.2051092	
**P(T<=t) one-tail**	**0.01468561**		**0.00568687**		**0.02608303**	
t Critical one-tail	2.91998558		2.91998558		2.91998558	
P(T<=t) two-tail	0.02937121		0.01137373		0.05216606	
t Critical two-tail	4.30265273		4.30265273		4.30265273	

**Neutral Condition**	**C18:3 Fatty Acid Content**		**C16:3 Fatty Acid Content**		**C18:1 Fatty Acid Content**	
**t-Test: Paired**	*ND*	*ND+1*	*ND*	*ND+1*	*ND*	*ND+1*
Mean	0.44589813	0.27223599	0.24584372	0.14127687	0.01225456	0.11654156
Variance	6.1253E-05	1.7569E-05	5.4401E-07	1.0028E-05	1.7324E-06	1.5404E-05
Observations	3	3	3	3	3	3
Pearson Correlation	0.96196892		-0.7628257		-0.0889749	
Df	2		2		2	
t Stat	75.8947298		48.1723165		-42.509156	
**P(T<=t) one-tail**	**8.6783E-05**		**0.00021532**		**0.00027647**	
t Critical one-tail	2.91998558		2.91998558		2.91998558	
P(T<=t) two-tail	0.00017357		0.00043065		0.00055294	
t Critical two-tail	4.30265273		4.30265273		4.30265273	

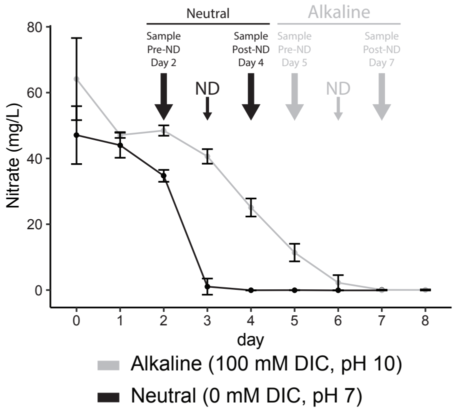

Timepoint	Illumina Sequences Generated (Rep 1)	Illumina Sequences Generated (Rep 2)	Illumina Sequences Generated (Rep 3)
Neutral Pre-ND	**64723168**	**51212730**	**N/A**
Neutral Post-ND	**91850794**	**78515846**	**108157912**
Alkaline Pre-ND	**85952882**	**142539990**	**154534478**
Alkaline Post-ND	**94814926**	**101506282**	**115114188**

Supplementary Figure S2: Sampling timepoints and sequencing coverage of this experiment.

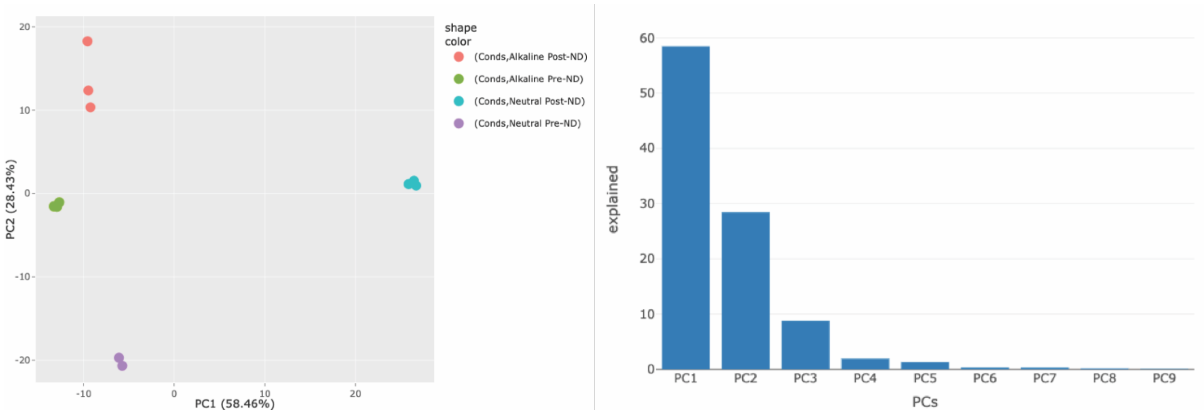

Supplementary Figure S3: Principal Component Analysis (PCA) analysis of RNA-sequencing data.

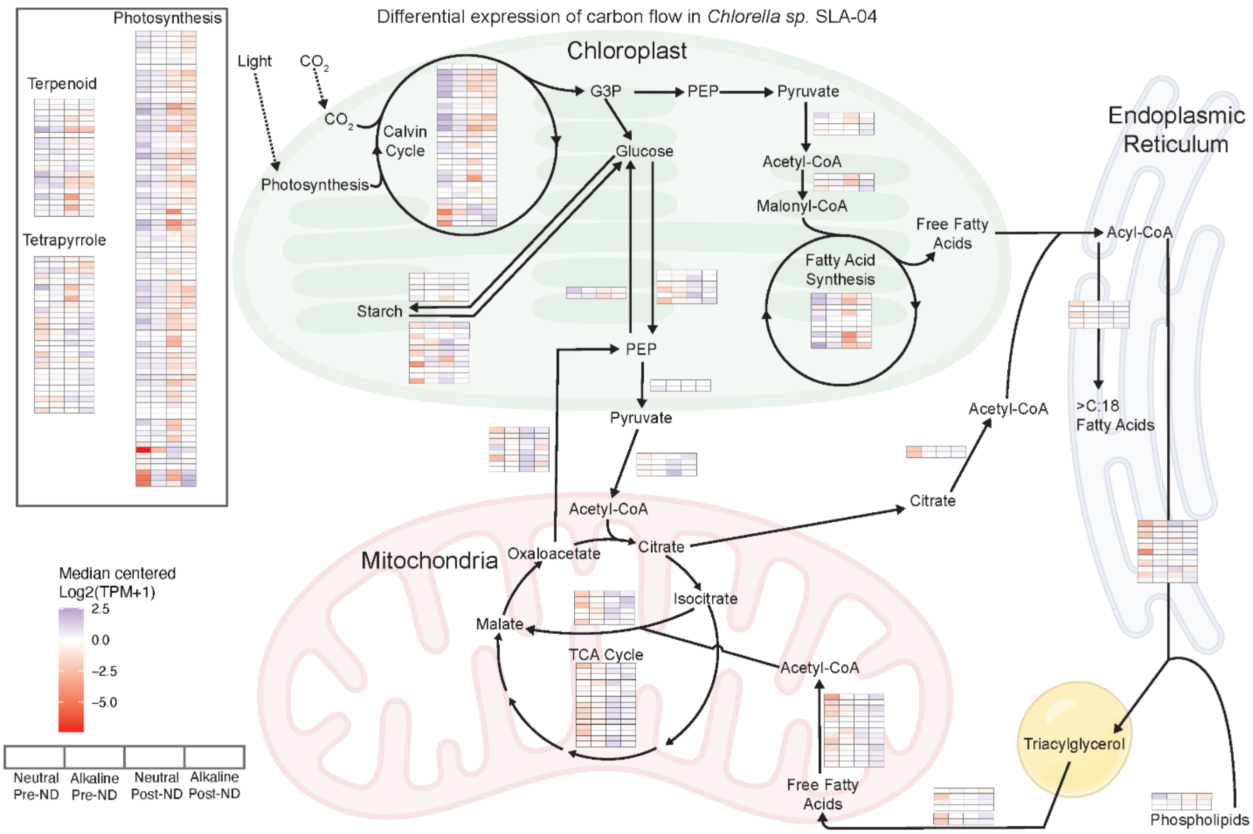

Supplementary Figure S4: Expanded version of Figure 3 in the manuscript providing more detailed differential expression of genes involved in carbon flow between growth conditions. Tables show the differential gene expression (DGE) of individual genes within the different functions of carbon metabolism. Each row represents a single gene, and each column represents a different condition (from left to right, Neutral Nitrogen-replete, Alkaline Nitrogen-replete, Neutral Nitrogen-deplete, and Alkaline Nitrogen-deplete). Red boxes represent downregulated genes, while blue boxes represent upregulated genes.

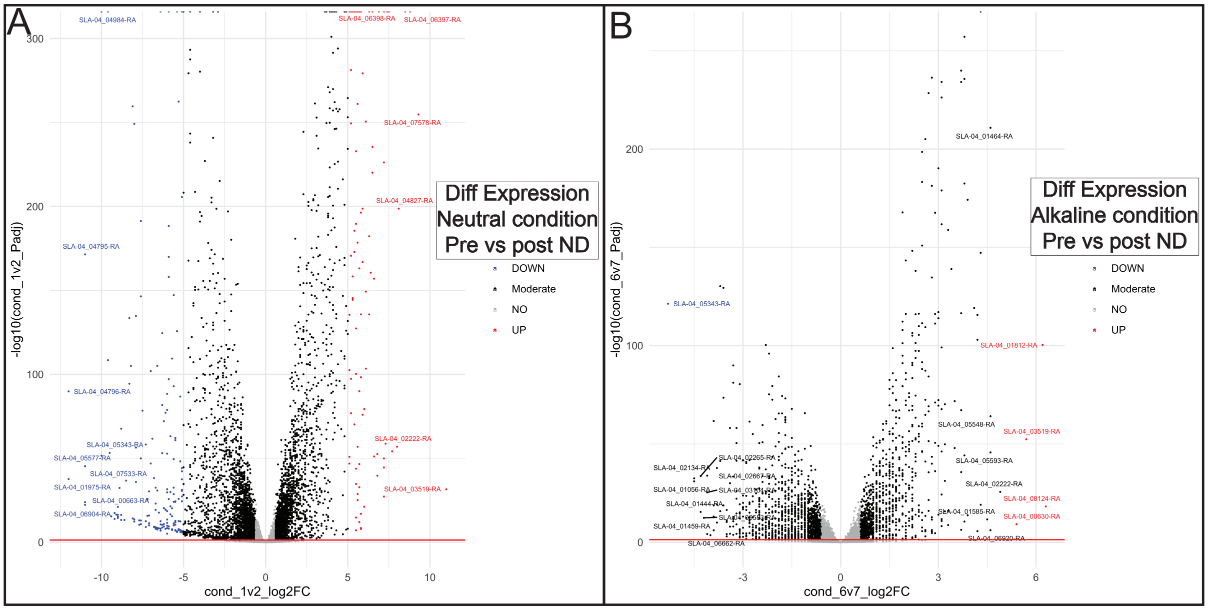

Supplementary Figure S5: Volcano plots showing differential gene expression (DGE) of SLA-04 pre- vs post-nitrogen depletion. (A) DGE under neutral conditions, comparing 24 hours before and after nitrogen depletion. (B) Shows the DGE of alkaline conditions, comparing 24 hours before and after nitrogen depletion. The x-axis is log2 of fold change, and the y-axis is -log10 of the p-value.

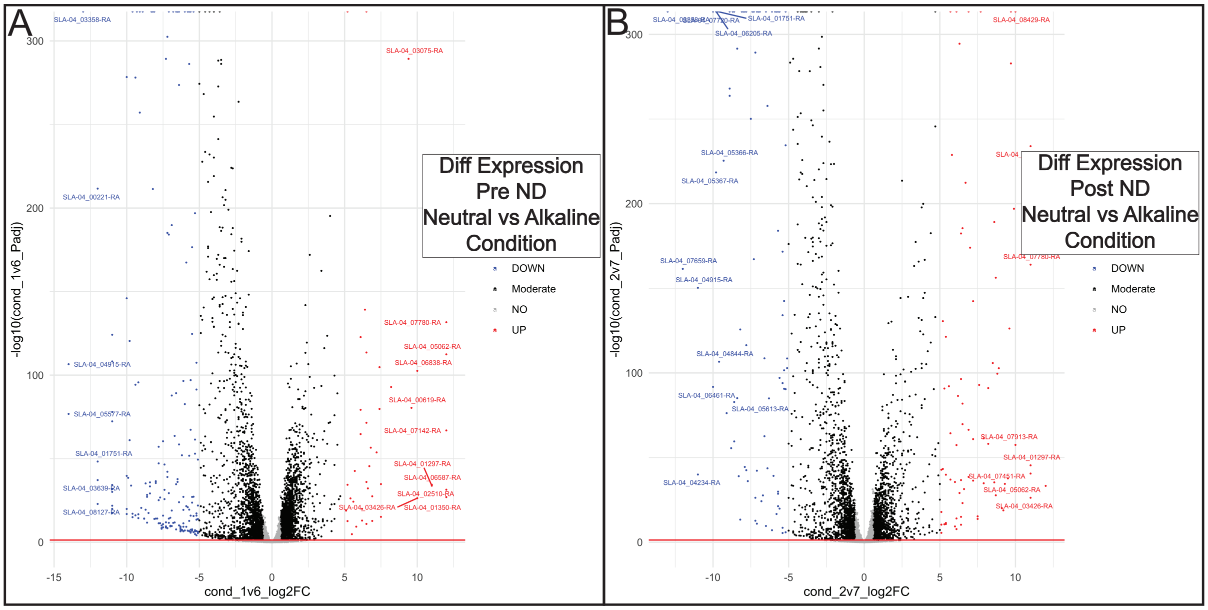

Supplementary Figure S6: Volcano plots showing differential gene expression (DGE) of SLA-04 grown in neutral vs alkaline media. (A) DGE comparing neutral versus alkaline cultures in nitrogen-replete conditions (B) Shows the DGE comparing neutral versus alkaline cultures in nitrogen-deplete conditions. The x-axis is log2 of fold change, and the y-axis is -log10 of the p-value.

Supplementary Table S3: Table of genes with statistically significant differential genes expression between pre- versus post-nitrogen depletion.

Gene ID	Average DGE	Annotation	Functional Category
SLA-04_03519-RA	-8.35	NDPK2 Nucleoside diphosphate kinase 2, chloroplastic (Nicotiana tabacum OX=4097)	Nucleotide metabolism
SLA-04_02222-RA	-6.45	OEP24A Outer envelope pore protein 24A, chloroplastic (Arabidopsis thaliana OX=3702)	not assigned
SLA-04_05548-RA	-6.15	CAH1 Carbonic anhydrase 1 (Chlamydomonas reinhardtii OX=3055)	Photosynthesis
SLA-04_04827-RA	-6	efeO Iron uptake system component EfeO (Shigella boydii serotype 4 (strain Sb227) OX=300268)	not assigned
SLA-04_05897-RA	-5.65	CYP38 Peptidyl-prolyl cis-trans isomerase CYP38, chloroplastic (Arabidopsis thaliana OX=3702)	Protein modification
SLA-04_00630-RA	-5.55	MAN1 Mannan endo-1,4-beta-mannosidase 1 (Oryza sativa subsp. japonica OX=39947)	Cell wall organization
SLA-04_06397-RA	-5.55	metE 5-methyltetrahydropteroyltriglutamate--homocysteine methyltransferase (Synechococcus sp. (strain JA-2-3B'a(2-13)) OX=321332)	not assigned
SLA-04_05896-RA	-5.55	TLP40 Peptidyl-prolyl cis-trans isomerase, chloroplastic (Spinacia oleracea OX=3562)	not assigned
SLA-04_06398-RA	-5.4	5-methyltetrahydropteroyltriglutamate--homocysteine methyltransferase (Chlamydomonas reinhardtii OX=3055)	not assigned
SLA-04_04008-RA	-4.95	unknown function	Photosynthesis
SLA-04_06418-RA	-4.95	FKBP16-3 Peptidyl-prolyl cis-trans isomerase FKBP16-3, chloroplastic (Arabidopsis thaliana OX=3702)	Protein modification
SLA-04_05086-RA	-4.85	SAMC2 Probable S-adenosylmethionine carrier 2, chloroplastic (Arabidopsis thaliana OX=3702)	Solute transport
SLA-04_02428-RA	-4.75	ASP1 Aspartate aminotransferase, mitochondrial (Arabidopsis thaliana OX=3702)	Nutrient uptake
SLA-04_02292-RA	-4.7	RPI3 Probable ribose-5-phosphate isomerase 3, chloroplastic (Arabidopsis thaliana OX=3702)	not assigned
SLA-04_02300-RA	-4.7	ISPD 2-C-methyl-D-erythritol 4-phosphate cytidylyltransferase, chloroplastic (Arabidopsis thaliana OX=3702)	Secondary metabolism
SLA-04_03993-RA	-4.65	Ppif Peptidyl-prolyl cis-trans isomerase F, mitochondrial (Mus musculus OX=10090)	Enzyme classification
SLA-04_01094-RA	-4.65	Sc5d Lathosterol oxidase (Rattus norvegicus OX=10116)	not assigned
SLA-04_07250-RA	-4.6	CIN2 Beta-fructofuranosidase, insoluble isoenzyme 2 (Oryza sativa subsp. indica OX=39946)	Enzyme classification
SLA-04_03682-RA	-4.6	GC1 Epimerase family protein SDR39U1 homolog, chloroplastic (Arabidopsis thaliana OX=3702)	not assigned
SLA-04_01455-RB	-4.4	NEK8 Serine/threonine-protein kinase Nek8 (Homo sapiens OX=9606)	not assigned
SLA-04_08304-RA	-4.35	unknown function	Coenzyme metabolism
SLA-04_01464-RA	-4.25	CSR1 Phosphatidylinositol transfer protein CSR1 (Ashbya gossypii (strain ATCC 10895 / CBS 109.51 / FGSC 9923 / NRRL Y-1056) OX=284811)	not assigned
SLA-04_01585-RA	-4.25	Cyp4x1 Cytochrome P450 4X1 (Rattus norvegicus OX=10116)	Enzyme classification
SLA-04_02716-RA	-4.25	fabD Malonyl CoA-acyl carrier protein transacylase (Bacillus subtilis (strain 168) OX=224308)	Lipid metabolism
SLA-04_00555-RA	-4.25	fabZ 3-hydroxyacyl-[acyl-carrier-protein] dehydratase FabZ (Bacillus velezensis (strain DSM 23117 / BGSC 10A6 / FZB42) OX=326423)	Lipid metabolism
SLA-04_05179-RA	-4.25	PSB27-1 Photosystem II repair protein PSB27-H1, chloroplastic (Arabidopsis thaliana OX=3702)	Photosynthesis
SLA-04_07251-RA	-4.2	CIN6 Beta-fructofuranosidase, insoluble isoenzyme 6 (Oryza sativa subsp. japonica OX=39947)	not assigned
SLA-04_05771-RA	-4.15	At3g63540 Thylakoid lumenal 19 kDa protein, chloroplastic (Arabidopsis thaliana OX=3702)	not assigned
SLA-04_05353-RA	-4.15	PETF Ferredoxin, chloroplastic (Chlamydomonas reinhardtii OX=3055)	Photosynthesis
SLA-04_01549-RA	-4.15	PPD5 PsbP domain-containing protein 5, chloroplastic (Arabidopsis thaliana OX=3702)	not assigned
SLA-04_01061-RA	-4.1	fdhC Probable formate transporter (Methanothermobacter thermautotrophicus OX=145262)	not assigned

SLA-04_04639-RA	-4.1	OsI_00941 GDT1-like protein 1, chloroplastic (Oryza sativa subsp. indica OX=39946)	Solute transport
SLA-04_06791-RA	-4.1	Triosephosphate isomerase, chloroplastic (Secale cereale OX=4550)	Enzyme classification
SLA-04_07203-RA	-4.05	MGD A Probable monogalactosyldiacylglycerol synthase, chloroplastic (Glycine max OX=3847)	Lipid metabolism
SLA-04_05593-RA	-4.05	TIC62 Protein TIC 62, chloroplastic (Pisum sativum OX=3888)	Photosynthesis
SLA-04_01928-RA	-4	SECY Preprotein translocase subunit SECY, chloroplastic (Pisum sativum OX=3888)	Protein translocation
SLA-04_07391-RA	-3.95	HEME2 Uroporphyrinogen decarboxylase 2, chloroplastic (Arabidopsis thaliana OX=3702)	Coenzyme metabolism
SLA-04_06507-RA	-3.95	rplF 50S ribosomal protein L6 (Gloeothece citriformis (strain PCC 7424) OX=65393)	Protein biosynthesis
SLA-04_02363-RA	-3.9	Setd4 SET domain-containing protein 4 (Mus musculus OX=10090)	not assigned
SLA-04_03544-RA	-3.85	PGK Phosphoglycerate kinase, chloroplastic (Volvox carteri OX=3067)	not assigned
SLA-04_01355-RA	-3.8	sll0418 2-methyl-6-phytyl-1,4-hydroquinone methyltransferase (Synechocystis sp. (strain PCC 6803 / Kazusa) OX=1111708)	not assigned
SLA-04_02273-RA	-3.75	PPD1 PsbP domain-containing protein 1, chloroplastic (Arabidopsis thaliana OX=3702)	not assigned
SLA-04_04404-RA	-3.75	PPL1 PsbP-like protein 1, chloroplastic (Arabidopsis thaliana OX=3702)	Photosynthesis
SLA-04_06422-RA	-3.6	ppa1 Soluble inorganic pyrophosphatase 1, chloroplastic (Chlamydomonas reinhardtii OX=3055)	Multi-process regulation
SLA-04_00455-RA	-3.6	CPX Oxygen-dependent coproporphyrinogen-III oxidase, chloroplastic (Glycine max OX=3847)	Coenzyme metabolism
SLA-04_01971-RA	-3.6	DVR Divinyl chlorophyllide a 8-vinyl-reductase, chloroplastic (Cucumis sativus OX=3659)	Coenzyme metabolism
SLA-04_08354-RA	-3.55	HEMC Porphobilinogen deaminase, chloroplastic (Oryza sativa subsp. japonica OX=39947)	Coenzyme metabolism
SLA-04_00646-RA	-3.55	Os09g0505700 Ribulose-phosphate 3-epimerase, cytoplasmic isoform (Oryza sativa subsp. japonica OX=39947)	Carbohydrate metabolism
SLA-04_05938-RA	-3.5	FRAAL3718 Putative S-adenosyl-L-methionine-dependent methyltransferase FRAAL3718 (Frankia alni (strain ACN14a) OX=326424)	not assigned
SLA-04_01993-RA	-3.5	IMPL1 Phosphatase IMPL1, chloroplastic (Arabidopsis thaliana OX=3702)	not assigned
SLA-04_06896-RA	-3.5	TATA Sec-independent protein translocase protein TATA, chloroplastic (Arabidopsis thaliana OX=3702)	Protein translocation
SLA-04_01799-RA	-3.45	FFC Signal recognition particle 54 kDa protein, chloroplastic (Arabidopsis thaliana OX=3702)	Protein translocation
SLA-04_06400-RA	-3.45	GDCST Aminomethyltransferase, mitochondrial (Mesembryanthemum crystallinum OX=3544)	Photosynthesis
SLA-04_06077-RA	-3.4	CHLM Magnesium protoporphyrin IX methyltransferase, chloroplastic (Arabidopsis thaliana OX=3702)	Coenzyme metabolism
SLA-04_06097-RC	-3.4	PORA Protochlorophyllide reductase, chloroplastic (Cucumis sativus OX=3659)	Coenzyme metabolism
SLA-04_03264-RA	-3.4	CPX Oxygen-dependent coproporphyrinogen-III oxidase, chloroplastic (Oryza sativa subsp. japonica OX=39947)	Enzyme classification
SLA-04_07504-RA	-3.35	CRTISO1 Prolycopene isomerase 1, chloroplastic (Oncidium hybrid cultivar OX=141207)	not assigned
SLA-04_00946-RA	-3.35	AKR2A Ankyrin repeat domain-containing protein 2A (Arabidopsis thaliana OX=3702)	Protein translocation
SLA-04_06404-RA	-3.3	ctpA C-terminal processing peptidase, chloroplastic (Tetradesmus obliquus OX=3088)	not assigned
SLA-04_01860-RA	-3.25	CLEB3J9 Thylakoid lumenal 29 kDa protein, chloroplastic (Solanum lycopersicum OX=4081)	not assigned
SLA-04_01463-RA	-3.25	PAT1 Anthranilate phosphoribosyltransferase, chloroplastic (Arabidopsis thaliana OX=3702)	Amino acid metabolism
SLA-04_02504-RA	-3.25	sll1737 Tic20 family protein (Synechocystis sp. (strain PCC 6803 / Kazusa) OX=1111708)	Protein translocation
SLA-04_08090-RA	-3.2	Fructose-bisphosphate aldolase, cytoplasmic isozyme (Spinacia oleracea OX=3562)	not assigned
SLA-04_07769-RA	-3.2	GAPA Glyceraldehyde-3-phosphate dehydrogenase A, chloroplastic (Spinacia oleracea OX=3562)	Photosynthesis
SLA-04_02169-RA	-3.2	TRPA1 Tryptophan synthase alpha chain (Arabidopsis thaliana OX=3702)	Amino acid metabolism

SLA-04_00800-RA	-3.15	LHCB5 Chlorophyll a-b binding protein CP26, chloroplastic (Arabidopsis thaliana OX=3702)	Photosynthesis
SLA-04_07646-RA	-3.15	PETE Plastocyanin, chloroplastic (Chlamydomonas reinhardtii OX=3055)	Photosynthesis
SLA-04_06521-RA	-3.15	asd Aspartate-semialdehyde dehydrogenase (Aquifex aeolicus (strain VF5) OX=224324)	Amino acid metabolism
SLA-04_06522-RA	-3.15	asd Aspartate-semialdehyde dehydrogenase (Synechocystis sp. (strain PCC 6803 / Kazusa) OX=1111708)	Amino acid metabolism
SLA-04_01182-RA	-3.15	DIT2-1 Dicarboxylate transporter 2.1, chloroplastic (Arabidopsis thaliana OX=3702)	Solute transport
SLA-04_01067-RA	-3.15	HST Homogentisate solanesyltransferase, chloroplastic (Chlamydomonas reinhardtii OX=3055)	not assigned
SLA-04_01679-RA	-3.1	VTE5 Phytol kinase 1, chloroplastic (Arabidopsis thaliana OX=3702)	not assigned
SLA-04_01990-RA	-3.05	At2g44920 Thylakoid lumenal 15 kDa protein 1, chloroplastic (Arabidopsis thaliana OX=3702)	not assigned
SLA-04_01680-RA	-3.05	VPS4 Vacuolar protein sorting-associated protein 4 (Saccharomyces cerevisiae (strain ATCC 204508 / S288c) OX=559292)	not assigned
SLA-04_01227-RA	-3	ABA4 Protein ABA DEFICIENT 4, chloroplastic (Arabidopsis thaliana OX=3702)	Secondary metabolism
SLA-04_06151-RA	-3	ALDCHL Fructose-bisphosphate aldolase 1, chloroplastic (Chlamydomonas reinhardtii OX=3055)	not assigned
SLA-04_01589-RA	-3	Caur_2266 Succinyl-CoA--D-citramalate CoA-transferase (Chloroflexus aurantiacus (strain ATCC 29366 / DSM 635 / J-10-fl) OX=324602)	not assigned
SLA-04_05621-RA	-3	CEST Protein CHLOROPLAST ENHANCING STRESS TOLERANCE, chloroplastic (Oryza sativa subsp. indica OX=39946)	Photosynthesis
SLA-04_01502-RA	-3	efp Elongation factor P (Synechococcus sp. (strain JA-2-3B'a(2-13)) OX=321332)	Protein biosynthesis
SLA-04_03443-RA	-2.95	alkJ Alcohol dehydrogenase [acceptor] (Pseudomonas oleovorans OX=301)	not assigned
SLA-04_03827-RA	-2.95	PRXIIE Peroxiredoxin-2E, chloroplastic (Arabidopsis thaliana OX=3702)	Redox homeostasis
SLA-04_01497-RA	-2.95	PSBP Oxygen-evolving enhancer protein 2, chloroplastic (Chlamydomonas reinhardtii OX=3055)	Photosynthesis
SLA-04_06003-RA	-2.9	CYP26-2 Peptidyl-prolyl cis-trans isomerase CYP26-2, chloroplastic (Arabidopsis thaliana OX=3702)	Secondary metabolism
SLA-04_00224-RA	-2.85	CHLD Magnesium-chelatase subunit ChlD, chloroplastic (Pisum sativum OX=3888)	Coenzyme metabolism
SLA-04_07137-RA	-2.8	CRTZ Beta-carotene 3-hydroxylase, chloroplastic (Fragment) (Haematococcus lacustris OX=44745)	Secondary metabolism
SLA-04_01528-RA	-2.8	PSAO Photosystem I subunit O (Arabidopsis thaliana OX=3702)	Photosynthesis
SLA-04_01551-RA	-2.75	At2g31400 Pentatricopeptide repeat-containing protein At2g31400, chloroplastic (Arabidopsis thaliana OX=3702)	not assigned
SLA-04_04973-RA	-2.75	PSAN Photosystem I reaction center subunit N, chloroplastic (Volvox carteri OX=3067)	Photosynthesis
SLA-04_08148-RA	-2.75	UROS Uroporphyrinogen-III synthase, chloroplastic (Arabidopsis thaliana OX=3702)	Coenzyme metabolism
SLA-04_06154-RA	-2.65	LIL3.1 Light-harvesting complex-like protein 3 isotype 1, chloroplastic (Arabidopsis thaliana OX=3702)	Photosynthesis
SLA-04_00647-RA	-2.65	PNSL5 Photosynthetic NDH subunit of lumenal location 5, chloroplastic (Arabidopsis thaliana OX=3702)	Enzyme classification
SLA-04_08213-RA	-2.65	CPX Oxygen-dependent coproporphyrinogen-III oxidase, chloroplastic (Glycine max OX=3847)	Coenzyme metabolism
SLA-04_03847-RA	-2.6	DDB_G0271742 UPF0548 protein (Dictyostelium discoideum OX=44689)	Solute transport
SLA-04_02007-RA	-2.55	bioC Malonyl-[acyl-carrier protein] O-methyltransferase (Paenibacillus sp. (strain JDR-2) OX=324057)	not assigned
SLA-04_06175-RA	-2.55	GSA Glutamate-1-semialdehyde 2,1-aminomutase, chloroplastic (Chlamydomonas reinhardtii OX=3055)	Coenzyme metabolism
SLA-04_00452-RA	-2.55	YLMG2 YlmG homolog protein 2, chloroplastic (Arabidopsis thaliana OX=3702)	not assigned
SLA-04_04611-RA	-2.5	CAS Calcium sensing receptor, chloroplastic (Arabidopsis thaliana OX=3702)	not assigned
SLA-04_02904-RA	-2.5	Chlorophyll a-b binding protein of LHCII type I, chloroplastic (Lemna gibba OX=4470)	Photosynthesis
SLA-04_04193-RA	-2.45	CTH1 Magnesium-protoporphyrin IX monomethyl ester [oxidative] cyclase 2, chloroplastic (Chlamydomonas reinhardtii OX=3055)	Coenzyme metabolism

SLA-04_00828-RA	-2.45	LHCA1 Chlorophyll a-b binding protein 6, chloroplastic (Arabidopsis thaliana OX=3702)	not assigned
SLA-04_06070-RA	-2.45	UPP Uracil phosphoribosyltransferase (Nicotiana tabacum OX=4097)	Nucleotide metabolism
SLA-04_06540-RA	-2.4	ALB3.1 Inner membrane ALBINO3-like protein 1, chloroplastic (Chlamydomonas reinhardtii OX=3055)	Protein translocation
SLA-04_04590-RA	-2.4	Dclre1a DNA cross-link repair 1A protein (Mus musculus OX=10090)	not assigned
SLA-04_00454-RB	-2.4	LTD Protein LHCP TRANSLOCATION DEFECT (Arabidopsis thaliana OX=3702)	not assigned
SLA-04_01567-RA	-2.4	(S)-coclaurine N-methyltransferase (Thalictrum flavum subsp. glaucum OX=150095)	not assigned
SLA-04_06267-RA	-2.4	chlI Magnesium-chelatase subunit ChlI (Cyanophora paradoxa OX=2762)	Coenzyme metabolism
SLA-04_03645-RA	-2.4	CHLP Geranylgeranyl diphosphate reductase, chloroplastic (Arabidopsis thaliana OX=3702)	Coenzyme metabolism
SLA-04_07705-RA	-2.4	Lhcb4 Chlorophyll a-b binding protein CP29 (Chlamydomonas reinhardtii OX=3055)	Photosynthesis
SLA-04_07643-RA	-2.35	CYN Cyanate hydratase (Phaeodactylum tricornutum (strain CCAP 1055/1) OX=556484)	Amino acid metabolism
SLA-04_01335-RA	-2.35	RCABP89 Chlorophyll a-b binding protein, chloroplastic (Oryza sativa subsp. japonica OX=39947)	Photosynthesis
SLA-04_02486-RA	-2.35	Chlorophyll a-b binding protein, chloroplastic (Petunia hybrida OX=4102)	not assigned
SLA-04_02410-RA	-2.35	PSAH Photosystem I reaction center subunit VI, chloroplastic (Chlamydomonas reinhardtii OX=3055)	Photosynthesis
SLA-04_03243-RA	-2.3	HEMB Delta-aminolevulinic acid dehydratase, chloroplastic (Chlamydomonas reinhardtii OX=3055)	Enzyme classification
SLA-04_01343-RA	-2.3	LHCA4 Chlorophyll a-b binding protein 4, chloroplastic (Arabidopsis thaliana OX=3702)	not assigned
SLA-04_02485-RA	-2.3	LHCA4 Chlorophyll a-b binding protein 4, chloroplastic (Arabidopsis thaliana OX=3702)	not assigned
SLA-04_02422-RA	-2.3	LUT2 Lycopene epsilon cyclase, chloroplastic (Arabidopsis thaliana OX=3702)	not assigned
SLA-04_07400-RA	-2.25	CAB8 Chlorophyll a-b binding protein 8, chloroplastic (Solanum lycopersicum OX=4081)	Photosynthesis
SLA-04_01827-RA	-2.25	PSBY Photosystem II core complex proteins psbY, chloroplastic (Spinacia oleracea OX=3562)	Photosynthesis
SLA-04_02375-RA	-2.2	ageIM Modification methylase AgeI (Thalassobius gelatinovorus OX=53501)	not assigned
SLA-04_06216-RA	-2.2	CHLH Magnesium-chelatase subunit ChlH, chloroplastic (Oryza sativa subsp. indica OX=39946)	Coenzyme metabolism
SLA-04_01075-RA	-2.15	CSP4 Cold shock domain-containing protein 4 (Arabidopsis thaliana OX=3702)	External stimuli response
SLA-04_00400-RA	-2.15	LHCA5 Photosystem I chlorophyll a/b-binding protein 5, chloroplastic (Arabidopsis thaliana OX=3702)	not assigned
SLA-04_01786-RA	-2.15	PPOX1 Protoporphyrinogen oxidase, chloroplastic (Oryza sativa subsp. japonica OX=39947)	Coenzyme metabolism
SLA-04_02757-RA	-2.15	hemE Uroporphyrinogen decarboxylase (Synechococcus sp. (strain ATCC 27264 / PCC 7002 / PR-6) OX=32049)	Coenzyme metabolism
SLA-04_06403-RA	-2.15	STR11 Rhodanese-like domain-containing protein 11, chloroplastic (Arabidopsis thaliana OX=3702)	not assigned
SLA-04_02993-RA	-2.1	PSAG Photosystem I reaction center subunit V, chloroplastic (Arabidopsis thaliana OX=3702)	Photosynthesis
SLA-04_01097-RA	2.1	ASZ1 Ankyrin repeat, SAM and basic leucine zipper domain-containing protein 1 (Neofelis nebulosa OX=61452)	not assigned
SLA-04_03102-RA	2.35	DGAT2A Diacylglycerol O-acyltransferase 2A (Umbelopsis ramanniana OX=41833)	not assigned
SLA-04_05737-RA	2.4	exo1 Exodeoxyribonuclease 1 (Schizosaccharomyces pombe (strain 972 / ATCC 24843) OX=284812)	not assigned
SLA-04_00425-RA	2.4	ZEU1 Thymidylate kinase (Arabidopsis thaliana OX=3702)	Nucleotide metabolism
SLA-04_02881-RA	2.4	erkB Extracellular signal-regulated kinase 2 (Dictyostelium discoideum OX=44689)	Multi-process regulation
SLA-04_07489-RA	2.45	At1g16220 Probable protein phosphatase 2C 6 (Arabidopsis thaliana OX=3702)	not assigned
SLA-04_02555-RA	2.5	HLJ1 Protein HLJ1 (Saccharomyces cerevisiae (strain ATCC 204508 / S288c) OX=559292)	not assigned

SLA-04_03372-RA	2.5	ODA7 Leucine-rich repeat-containing protein ODA7 (Chlamydomonas reinhardtii OX=3055)	not assigned
SLA-04_05523-RA	2.5	RCL1 RNA 3'-terminal phosphate cyclase-like protein (Bos taurus OX=9913)	not assigned
SLA-04_00908-RA	2.55	AURKA Aurora kinase A (Bos taurus OX=9913)	Enzyme classification
SLA-04_06780-RA	2.55	RAD54 DNA repair and recombination protein RAD54 (Oryza sativa subsp. japonica OX=39947)	not assigned
SLA-04_02323-RA	2.55	SPBC1711.16 Uncharacterized WD repeat-containing protein C17D11.16 (Schizosaccharomyces pombe (strain 972 / ATCC 24843) OX=284812)	not assigned
SLA-04_02512-RA	2.65	SPAC3F10.06c Uncharacterized protein C3F10.06c (Schizosaccharomyces pombe (strain 972 / ATCC 24843) OX=284812)	not assigned
SLA-04_00412-RA	2.65	RH20 DEAD-box ATP-dependent RNA helicase 20 (Arabidopsis thaliana OX=3702)	not assigned
SLA-04_05674-RA	2.65	SAT17 Taurine hydroxylase-like protein SAT17 (Stachybotrys chartarum (strain CBS 109288 / IBT 7711) OX=1280523)	not assigned
SLA-04_07089-RA	2.75	obr1 P25 protein (Schizosaccharomyces pombe (strain 972 / ATCC 24843) OX=284812)	not assigned
SLA-04_02691-RA	2.75	RFS2 Probable galactinol--sucrose galactosyltransferase 2 (Arabidopsis thaliana OX=3702)	not assigned
SLA-04_04752-RA	2.8	Os03g0728800 DEAD-box ATP-dependent RNA helicase 16 (Oryza sativa subsp. japonica OX=39947)	not assigned
SLA-04_06935-RA	2.8	pus10 Putative tRNA pseudouridine synthase Pus10 (Xenopus laevis OX=8355)	RNA processing
SLA-04_05716-RA	2.85	rumi O-glucosyltransferase rumi (Drosophila pseudoobscura OX=46245)	not assigned
SLA-04_06254-RA	2.9	atg26 Sterol 3-beta-glucosyltransferase (Aspergillus clavatus (strain ATCC 1007 / CBS 513.65 / DSM 816 / NCTC 3887 / NRRL 1) OX=344612)	not assigned
SLA-04_03497-RA	2.9	EMB2001 GTP-binding protein At2g22870 (Arabidopsis thaliana OX=3702)	not assigned
SLA-04_02094-RA	2.9	Tbc1d2 TBC1 domain family member 2A (Mus musculus OX=10090)	not assigned
SLA-04_01706-RA	2.95	SGT1 Peptidyl serine alpha-galactosyltransferase (Chlamydomonas reinhardtii OX=3055)	Cell wall organization
SLA-04_03447-RA	3	LDL1 Lysine-specific histone demethylase 1 homolog 1 (Arabidopsis thaliana OX=3702)	not assigned
SLA-04_05807-RA	3	RAD51D DNA repair protein RAD51 homolog 4 (Arabidopsis thaliana OX=3702)	DNA damage response
SLA-04_02624-RA	3	TRANK1 TPR and ankyrin repeat-containing protein 1 (Homo sapiens OX=9606)	not assigned
SLA-04_04262-RA	3.1	Wdr78 WD repeat-containing protein 78 (Rattus norvegicus OX=10116)	not assigned
SLA-04_05965-RA	3.15	ackA Acetate kinase (Desulfovibrio alaskensis (strain G20) OX=207559)	not assigned
SLA-04_03340-RA	3.15	DDB_G0277071 Probable serine/threonine-protein kinase DDB_G0277071 (Dictyostelium discoideum OX=44689)	Enzyme classification
SLA-04_05290-RA	3.15	Fam221b Protein FAM221B (Rattus norvegicus OX=10116)	not assigned
SLA-04_05719-RA	3.2	AVT6E Amino acid transporter AVT6E (Arabidopsis thaliana OX=3702)	not assigned
SLA-04_02226-RA	3.2	FSM Chromatin assembly factor 1 subunit FSM (Oryza sativa subsp. japonica OX=39947)	Chromatin organization
SLA-04_06790-RA	3.2	ORC4 Origin of replication complex subunit 4 (Arabidopsis thaliana OX=3702)	Cell division
SLA-04_05195-RA	3.2	POLE2 DNA polymerase epsilon subunit 2 (Gallus gallus OX=9031)	not assigned
SLA-04_00948-RA	3.25	At1g60750 Probable aldo-keto reductase 6 (Arabidopsis thaliana OX=3702)	not assigned
SLA-04_08285-RA	3.25	FAP3 Fatty-acid-binding protein 3, chloroplastic (Arabidopsis thaliana OX=3702)	not assigned
SLA-04_04267-RA	3.25	pfl Formate acetyltransferase (Clostridium pasteurianum OX=1501)	not assigned
SLA-04_05194-RA	3.3	DPB2 DNA polymerase epsilon subunit B (Arabidopsis thaliana OX=3702)	Cell division
SLA-04_01577-RA	3.3	HAC1 Protein HIGH ARSENIC CONTENT 1, mitochondrial (Arabidopsis thaliana OX=3702)	not assigned

SLA-04_04954-RA	3.3	TOUSLED Serine/threonine-protein kinase TOUSLED (Arabidopsis thaliana OX=3702)	Protein modification
SLA-04_07685-RA	3.35	ANP2 Mitogen-activated protein kinase kinase kinase 2 (Arabidopsis thaliana OX=3702)	Enzyme classification
SLA-04_08008-RA	3.35	ROA2 DNA replication licensing factor MCM3 homolog 2 (Zea mays OX=4577)	Cell division
SLA-04_01411-RA	3.35	mad2l1-1 Mitotic spindle assembly checkpoint protein MAD2A (Dictyostelium discoideum OX=44689)	not assigned
SLA-04_00799-RA	3.45	hndD NADP-reducing hydrogenase subunit HndD (Desulfovibrio fructosivorans OX=878)	not assigned
SLA-04_01333-RA	3.5	BTR1 Protein BTR1 (Arabidopsis thaliana OX=3702)	not assigned
SLA-04_00747-RA	3.5	HSP17.6-L 17.6 kDa class I heat shock protein (Glycine max OX=3847)	not assigned
SLA-04_06343-RA	3.5	ODA9 Dynein, 78 kDa intermediate chain, flagellar outer arm (Chlamydomonas reinhardtii OX=3055)	Cytoskeleton organization
SLA-04_07765-RA	3.55	GLX1 Lactoylglutathione lyase GLX1 (Arabidopsis thaliana OX=3702)	not assigned
SLA-04_01515-RA	3.55	NDC80 Kinetochore protein NDC80 homolog (Gallus gallus OX=9031)	Cell division
SLA-04_02206-RA	3.55	GDHA Glutamate dehydrogenase A (Nicotiana plumbaginifolia OX=4092)	Nutrient uptake
SLA-04_04090-RA	3.6	DFR Bifunctional dihydroflavonol 4-reductase/flavanone 4-reductase (Pyrus communis OX=23211)	not assigned
SLA-04_06383-RA	3.6	KIN12F Kinesin-like protein KIN-12F (Oryza sativa subsp. japonica OX=39947)	not assigned
SLA-04_01460-RA	3.6	OsI_36121 DNA replication licensing factor MCM2 (Oryza sativa subsp. indica OX=39946)	Cell division
SLA-04_00840-RA	3.65	MCM7 DNA replication licensing factor MCM7 (Oryza sativa subsp. japonica OX=39947)	not assigned
SLA-04_05798-RA	3.7	NHX6 Sodium/hydrogen exchanger 6 (Arabidopsis thaliana OX=3702)	Solute transport
SLA-04_01940-RA	3.75	DDB_G0291133 Probable protein kinase DDB_G0291133 (Dictyostelium discoideum OX=44689)	Enzyme classification
SLA-04_02556-RA	3.8	GOLS2 Galactinol synthase 2 (Arabidopsis thaliana OX=3702)	not assigned
SLA-04_05191-RA	3.85	Os05g0235800 DNA replication licensing factor MCM6 (Oryza sativa subsp. japonica OX=39947)	not assigned
SLA-04_05975-RA	3.85	rnaseh2b Ribonuclease H2 subunit B (Xenopus laevis OX=8355)	Cell division
SLA-04_01393-RA	3.9	Os01g0868300 DNA polymerase alpha catalytic subunit (Oryza sativa subsp. japonica OX=39947)	Cell division
SLA-04_02148-RA	3.9	SUVH4 Histone-lysine N-methyltransferase, H3 lysine-9 specific SUVH4 (Arabidopsis thaliana OX=3702)	not assigned
SLA-04_03446-RA	3.95	NIC2 Nicotinamidase 2 (Arabidopsis thaliana OX=3702)	Coenzyme metabolism
SLA-04_05190-RA	3.95	MCM6 DNA replication licensing factor MCM6 (Arabidopsis thaliana OX=3702)	not assigned
SLA-04_08326-RA	3.95	SMC2-1 Structural maintenance of chromosomes protein 2-1 (Arabidopsis thaliana OX=3702)	Cell division
SLA-04_07143-RA	4	At4g14490 FHA domain-containing protein At4g14490 (Arabidopsis thaliana OX=3702)	Cell division
SLA-04_07796-RA	4.05	MYB3R5 Transcription factor MYB3R-5 (Arabidopsis thaliana OX=3702)	not assigned
SLA-04_03318-RA	4.05	SQD2 Sulfoquinovosyl transferase SQD2 (Arabidopsis thaliana OX=3702)	Lipid metabolism
SLA-04_05478-RA	4.05	CBSX3 CBS domain-containing protein CBSX3, mitochondrial (Arabidopsis thaliana OX=3702)	not assigned
SLA-04_02134-RA	4.15	CPK1 Calcium-dependent protein kinase 1 (Arabidopsis thaliana OX=3702)	Enzyme classification
SLA-04_00798-RA	4.15	NAD11 NADH-ubiquinone oxidoreductase 75 kDa subunit (Reclinomonas americana OX=48483)	not assigned
SLA-04_02009-RA	4.25	AZG1 Adenine/guanine permease AZG1 (Arabidopsis thaliana OX=3702)	Nucleotide metabolism
SLA-04_00766-RA	4.25	CHTF8 Chromosome transmission fidelity protein 8 homolog (Homo sapiens OX=9606)	not assigned
SLA-04_07342-RA	4.25	PXG1 Peroxygenase 1 (Arabidopsis thaliana OX=3702)	Lipid metabolism
SLA-04_02503-RA	4.3	MCM5 DNA replication licensing factor MCM5 (Oryza sativa subsp. japonica OX=39947)	not assigned

SLA-04_05159-RA	4.35	ARC5 Dynamin-like protein ARC5 (Arabidopsis thaliana OX=3702)	Cell division
SLA-04_01293-RA	4.4	CDCA7L Cell division cycle-associated 7-like protein (Homo sapiens OX=9606)	not assigned
SLA-04_03371-RA	4.4	ODA7 Leucine-rich repeat-containing protein ODA7 (Chlamydomonas reinhardtii OX=3055)	not assigned
SLA-04_04145-RA	4.4	unknown function	RNA processing
SLA-04_05443-RA	4.45	POL2A DNA polymerase epsilon catalytic subunit A (Arabidopsis thaliana OX=3702)	Cell division
SLA-04_03860-RA	4.5	incenp-b Inner centromere protein B (Xenopus laevis OX=8355)	not assigned
SLA-04_01459-RA	4.5	WEE1 Wee1-like protein kinase (Oryza sativa subsp. japonica OX=39947)	Cell division
SLA-04_07760-RA	4.55	NAT2 Nucleobase-ascorbate transporter 2 (Arabidopsis thaliana OX=3702)	Solute transport
SLA-04_01727-RA	4.6	NEK3 Serine/threonine-protein kinase Nek3 (Homo sapiens OX=9606)	Enzyme classification
SLA-04_00142-RA	4.8	alxA Alternative oxidase, mitochondrial (Emericella nidulans (strain FGSC A4 / ATCC 38163 / CBS 112.46 / NRRL 194 / M139) OX=227321)	not assigned
SLA-04_03910-RA	4.9	MFSD6 Major facilitator superfamily domain-containing protein 6 (Sus scrofa OX=9823)	not assigned
SLA-04_02667-RA	4.95	CDC45 Cell division control protein 45 homolog (Homo sapiens OX=9606)	Cell division
SLA-04_07268-RA	5.15	AAO2 Indole-3-acetaldehyde oxidase (Arabidopsis thaliana OX=3702)	not assigned
SLA-04_02138-RA	5.3	MSD1 Superoxide dismutase [Mn] 1, mitochondrial (Arabidopsis thaliana OX=3702)	Redox homeostasis
SLA-04_08060-RA	5.35	CURT1C Protein CURVATURE THYLAKOID 1C, chloroplastic (Arabidopsis thaliana OX=3702)	Photosynthesis
SLA-04_02943-RA	5.55	TPH1 Tryptophan 5-hydroxylase 1 (Oryctolagus cuniculus OX=9986)	Amino acid metabolism
SLA-04_06583-RA	5.65	BT_0258 Putative glucosamine-6-phosphate deaminase-like protein BT_0258 (Bacteroides thetaiotaomicron (strain ATCC 29148 / DSM 2079 / NCTC 10582	not assigned
SLA-04_01056-RA	5.7	uapA Uric acid-xanthine permease (Emericella nidulans (strain FGSC A4 / ATCC 38163 / CBS 112.46 / NRRL 194 / M139) OX=227321)	not assigned
SLA-04_00479-RA	5.8	CCD8 Carotenoid cleavage dioxygenase 8, chloroplastic (Arabidopsis thaliana OX=3702)	not assigned
SLA-04_03252-RA	5.85	SKOR Potassium channel SKOR (Arabidopsis thaliana OX=3702)	Solute transport
SLA-04_08414-RA	5.95	ODA2 Dynein gamma chain, flagellar outer arm (Chlamydomonas reinhardtii OX=3055)	Cytoskeleton organization
SLA-04_01975-RA	7.65	BAT1 Amino-acid permease BAT1 (Arabidopsis thaliana OX=3702)	Solute transport

Supplementary Table S4: Table of genes with statistically significant differential genes expression between neutral versus alkaline cultivation conditions

Gene ID	Average DGE	Annotation	Functional Category
SLA-04_05062-RA	-12	GINT1 Glucosamine inositolphosphorylceramide transferase 1 (Oryza sativa subsp. japonica OX=39947)	Lipid metabolism
SLA-04_02510-RA	-10.55	FRAS1 Extracellular matrix protein FRAS1 (Homo sapiens OX=9606)	not assigned
SLA-04_03426-RA	-10.5	SULTR2 Proton/sulfate cotransporter 2 (Chlamydomonas reinhardtii OX=3055)	Solute transport
SLA-04_07142-RA	-10.35	NRAMP2 Metal transporter Nramp2 (Oryza sativa subsp. japonica OX=39947)	not assigned
SLA-04_03075-RA	-9.55	CID11 Polyadenylate-binding protein-interacting protein 11 (Arabidopsis thaliana OX=3702)	not assigned
SLA-04_01350-RA	-9	ftr1 High affinity iron permease 1 (Rhizopus delemar (strain RA 99-880 / ATCC MYA-4621 / FGSC 9543 / NRRL 43880) OX=246409)	Nutrient uptake
SLA-04_06838-RA	-8.6	Hephaestin-like protein (Acropora millepora OX=45264)	Nutrient uptake
SLA-04_04453-RA	-7.7	PTPRJ Receptor-type tyrosine-protein phosphatase eta (Gallus gallus OX=9031)	not assigned
SLA-04_00948-RA	-7.4	At1g60750 Probable aldo-keto reductase 6 (Arabidopsis thaliana OX=3702)	not assigned
SLA-04_07701-RA	-7	rtn4ip1 Reticulon-4-interacting protein 1 homolog, mitochondrial (Danio rerio OX=7955)	not assigned
SLA-04_08285-RA	-6.95	FAP3 Fatty-acid-binding protein 3, chloroplastic (Arabidopsis thaliana OX=3702)	not assigned
SLA-04_07612-RA	-6.65	mtcA1 Beta-carbonic anhydrase 1 (Mycobacterium tuberculosis (strain ATCC 25618 / H37Rv) OX=83332)	Photosynthesis
SLA-04_00007-RA	-6.5	APK3 Adenylyl-sulfate kinase 3 (Arabidopsis thaliana OX=3702)	Coenzyme metabolism
SLA-04_02085-RA	-6.45	BASS4 Probable sodium/metabolite cotransporter BASS4, chloroplastic (Oryza sativa subsp. japonica OX=39947)	Solute transport
SLA-04_03940-RA	-6.25	Wnk4 Serine/threonine-protein kinase WNK4 (Mus musculus OX=10090)	Enzyme classification
SLA-04_07913-RA	-6.15	lsd18 Putative epoxidase LasC (Streptomyces lasaliensis OX=324833)	not assigned
SLA-04_03422-RA	-5.9	CLIPB4 CLIP domain-containing serine protease 14D (Anopheles gambiae OX=7165)	not assigned
SLA-04_02308-RA	-5.2	GIT1 Glycerophosphoinositol permease 1 (Candida albicans (strain SC5314 / ATCC MYA-2876) OX=237561)	Solute transport
SLA-04_01551-RA	-5.1	At2g31400 Pentatricopeptide repeat-containing protein At2g31400, chloroplastic (Arabidopsis thaliana OX=3702)	not assigned
SLA-04_01782-RA	-5.05	DMBT1 Deleted in malignant brain tumors 1 protein (Homo sapiens OX=9606)	Enzyme classification
SLA-04_03452-RA	-5.05	pho-4 Phosphate-repressible phosphate permease pho-4 (Neurospora crassa (strain ATCC 24698 / 74-OR23-1A / CBS 708.71 / DSM 1257 / FGSC 987) OX=36	Solute transport
SLA-04_06237-RA	-4.55	BC10 Glycosyltransferase BC10 (Oryza sativa subsp. japonica OX=39947)	not assigned
SLA-04_08428-RA	-4.35	ZIP3 Zinc transporter 3 (Oryza sativa subsp. japonica OX=39947)	Solute transport
SLA-04_02956-RA	-4.25	AMT1-1 Ammonium transporter 1 member 1 (Arabidopsis thaliana OX=3702)	Solute transport
SLA-04_00872-RA	-4.25	BZ1 Anthocyanidin 3-O-glucosyltransferase (Zea mays OX=4577)	Enzyme classification
SLA-04_01056-RA	-3.95	uapA Uric acid-xanthine permease (Emericella nidulans (strain FGSC A4 / ATCC 38163 / CBS 112.46 / NRRL 194 / M139) OX=227321)	not assigned
SLA-04_02185-RA	-3.9	FMO1 Probable flavin-containing monooxygenase 1 (Arabidopsis thaliana OX=3702)	not assigned
SLA-04_07121-RA	-3.9	NUP58 Nuclear pore complex protein NUP58 (Arabidopsis thaliana OX=3702)	Protein homeostasis
SLA-04_03834-RA	-3.65	hcp Hydroxylamine reductase (Burkholderia vietnamiensis (strain G4 / LMG 22486) OX=269482)	not assigned
SLA-04_01728-RA	-3.65	Nek1 Serine/threonine-protein kinase Nek1 (Mus musculus OX=10090)	Enzyme classification
SLA-04_03444-RB	-3.6	p80 Protein P80 (Dictyostelium discoideum OX=44689)	not assigned
SLA-04_03833-RA	-3.2	hcp Hydroxylamine reductase (Shewanella amazonensis (strain ATCC BAA-1098 / SB2B) OX=326297)	not assigned

SLA-04_02218-RA	-3.2	P4H7 Probable prolyl 4-hydroxylase 7 (Arabidopsis thaliana OX=3702)	Protein modification
SLA-04_00199-RA	-3.05	AIL5 AP2-like ethylene-responsive transcription factor AIL5 (Arabidopsis thaliana OX=3702)	not assigned
SLA-04_06909-RA	-3.05	drkA Probable serine/threonine-protein kinase drkA (Dictyostelium discoideum OX=44689)	Multi-process regulation
SLA-04_01080-RA	-2.95	slc38a7 Putative sodium-coupled neutral amino acid transporter 7 (Xenopus laevis OX=8355)	not assigned
SLA-04_00254-RA	-2.8	ANK1 Ankyrin-1 (Homo sapiens OX=9606)	not assigned
SLA-04_05996-RA	-2.8	PGDH1 D-3-phosphoglycerate dehydrogenase 1, chloroplastic (Arabidopsis thaliana OX=3702)	Amino acid metabolism
SLA-04_07568-RA	-2.75	MAP3K11 Mitogen-activated protein kinase kinase kinase 11 (Homo sapiens OX=9606)	Enzyme classification
SLA-04_05716-RA	-2.7	rumi O-glucosyltransferase rumi (Drosophila pseudoobscura pseudoobscura OX=46245)	not assigned
SLA-04_02849-RA	-2.65	BOU Mitochondrial carnitine/acylcarnitine carrier-like protein (Arabidopsis thaliana OX=3702)	Solute transport
SLA-04_03703-RA	-2.55	SYD Chromatin structure-remodeling complex protein SYD (Arabidopsis thaliana OX=3702)	not assigned
SLA-04_07566-RA	-2.5	aq_1088 Uncharacterized protein aq_1088 (Aquifex aeolicus (strain VF5) OX=224324)	not assigned
SLA-04_07963-RA	-2.4	Slc8b1 Mitochondrial sodium/calcium exchanger protein (Mus musculus OX=10090)	Solute transport
SLA-04_04137-RA	-2.35	PTAC14 Protein PLASTID TRANSCRIPTIONALLY ACTIVE 14 (Arabidopsis thaliana OX=3702)	not assigned
SLA-04_05677-RA	-2.25	Rad54l DNA repair and recombination protein RAD54-like (Mus musculus OX=10090)	not assigned
SLA-04_04397-RB	-2.05	AP17 AP-2 complex subunit sigma (Arabidopsis thaliana OX=3702)	Protein modification
SLA-04_07786-RA	2.1	APUM3 Pumilio homolog 3 (Arabidopsis thaliana OX=3702)	not assigned
SLA-04_07002-RA	2.15	BUB1 Mitotic checkpoint serine/threonine-protein kinase BUB1 (Arabidopsis thaliana OX=3702)	Enzyme classification
SLA-04_06950-RA	2.15	SETH3 Probable arabinose 5-phosphate isomerase (Arabidopsis thaliana OX=3702)	Cell wall organization
SLA-04_04766-RA	2.2	DDB_G0281745 Probable serine/threonine-protein kinase DDB_G0281745 (Dictyostelium discoideum OX=44689)	Enzyme classification
SLA-04_01383-RA	2.2	PROC1 Pyrroline-5-carboxylate reductase (Arabidopsis thaliana OX=3702)	Amino acid metabolism
SLA-04_03964-RA	2.25	PRY1 Protein PRY1 (Saccharomyces cerevisiae (strain ATCC 204508 / S288c) OX=559292)	Phytohormone action
SLA-04_03922-RA	2.25	unknown function	Solute transport
SLA-04_03802-RA	2.3	At3g26670 Probable magnesium transporter NIPA8 (Arabidopsis thaliana OX=3702)	Solute transport
SLA-04_06811-RA	2.3	ddiA Ribonuclease DdI (Dictyostelium discoideum OX=44689)	RNA processing
SLA-04_04636-RB	2.3	SPC25 Kinetochore protein Spc25 (Bos taurus OX=9913)	Cell division
SLA-04_01043-RA	2.35	gap3 Glyceraldehyde-3-phosphate dehydrogenase 3 (Nostoc sp. (strain PCC 7120 / SAG 25.82 / UTEX 2576) OX=103690)	Enzyme classification
SLA-04_07031-RA	2.35	PGPS2 CDP-diacylglycerol--glycerol-3-phosphate 3-phosphatidyltransferase 2 (Arabidopsis thaliana OX=3702)	not assigned
SLA-04_01097-RA	2.4	ASZ1 Ankyrin repeat, SAM and basic leucine zipper domain-containing protein 1 (Neofelis nebulosa OX=61452)	not assigned
SLA-04_04347-RA	2.4	Epn2 Epsin-2 (Rattus norvegicus OX=10116)	not assigned
SLA-04_05060-RA	2.45	CAO Chlorophyllide a oxygenase, chloroplastic (Chlamydomonas reinhardtii OX=3055)	Coenzyme metabolism
SLA-04_04596-RA	2.45	KIN7D Kinesin-like protein KIN-7D, mitochondrial (Arabidopsis thaliana OX=3702)	not assigned
SLA-04_06554-RA	2.5	hh Protein hedgehog (Drosophila virilis OX=7244)	not assigned
SLA-04_06641-RA	2.5	SBP2 Squamosa promoter-binding protein 2 (Antirrhinum majus OX=4151)	RNA biosynthesis
SLA-04_01558-RA	2.5	speA Biosynthetic arginine decarboxylase (Nostoc sp. (strain PCC 7120 / SAG 25.82 / UTEX 2576) OX=103690)	Polyamine metabolism
SLA-04_07382-RA	2.55	PDPK1 3-phosphoinositide-dependent protein kinase 1 (Arabidopsis thaliana OX=3702)	Protein modification

SLA-04_07755-RA	2.65	ODA1 Outer dynein arm protein 1 (Chlamydomonas reinhardtii OX=3055)	not assigned
SLA-04_06178-RA	2.7	Retrovirus-related Pol polyprotein from transposon TNT 1-94 (Nicotiana tabacum OX=4097)	not assigned
SLA-04_06929-RA	2.8	At5g53320 Probable inactive receptor kinase At5g53320 (Arabidopsis thaliana OX=3702)	not assigned
SLA-04_02074-RA	2.85	BC10 Glycosyltransferase BC10 (Oryza sativa subsp. japonica OX=39947)	not assigned
SLA-04_04963-RA	2.85	bp26 26 kDa periplasmic immunogenic protein (Brucella suis biovar 1 (strain 1330) OX=204722)	not assigned
SLA-04_07120-RA	2.85	SERPINA3-1 Serpin A3-1 (Bos taurus OX=9913)	Protein homeostasis
SLA-04_01685-RA	2.85	TPPP2 Tubulin polymerization-promoting protein family member 2 (Homo sapiens OX=9606)	not assigned
SLA-04_00896-RA	2.9	GALT5 Hydroxyproline O-galactosyltransferase GALT5 (Arabidopsis thaliana OX=3702)	not assigned
SLA-04_05404-RA	2.9	At5g01750 Protein LURP-one-related 15 (Arabidopsis thaliana OX=3702)	not assigned
SLA-04_01240-RA	3	SPL4 Squamosa promoter-binding-like protein 4 (Arabidopsis thaliana OX=3702)	RNA biosynthesis
SLA-04_02442-RA	3	ugt3a1 UDP-glucuronosyltransferase 3A1 (Xenopus laevis OX=8355)	Enzyme classification
SLA-04_07489-RA	3.05	At1g16220 Probable protein phosphatase 2C 6 (Arabidopsis thaliana OX=3702)	not assigned
SLA-04_01243-RA	3.05	tagA Serine protease/ABC transporter B family protein tagA (Dictyostelium discoideum OX=44689)	not assigned
SLA-04_02721-RA	3.1	ecaA Carbonic anhydrase (Nostoc sp. (strain PCC 7120 / SAG 25.82 / UTEX 2576) OX=103690)	Photosynthesis
SLA-04_01931-RA	3.1	RECQL5 ATP-dependent DNA helicase Q-like 5 (Arabidopsis thaliana OX=3702)	not assigned
SLA-04_06499-RA	3.15	KIN7I Kinesin-like protein KIN-7I (Oryza sativa subsp. japonica OX=39947)	Cytoskeleton organization
SLA-04_02581-RA	3.15	unknown function	Vesicle trafficking
SLA-04_08002-RA	3.2	Histone H4 (Chelidonium majus OX=71251)	Chromatin organization
SLA-04_04070-RA	3.25	fabG 3-oxoacyl-[acyl-carrier-protein] reductase FabG (Thermotoga maritima (strain ATCC 43589 / MSB8 / DSM 3109 / JCM 10099) OX=243274)	not assigned
SLA-04_07524-RA	3.25	GGP1 Gamma-glutamyl peptidase 1 (Arabidopsis thaliana OX=3702)	Protein homeostasis
SLA-04_04410-RA	3.25	SPATA20 Spermatogenesis-associated protein 20 (Homo sapiens OX=9606)	not assigned
SLA-04_02471-RA	3.3	CNR11 Cell number regulator 11 (Zea mays OX=4577)	not assigned
SLA-04_07102-RA	3.3	MRS2-7 Magnesium transporter MRS2-7 (Arabidopsis thaliana OX=3702)	Solute transport
SLA-04_04687-RA	3.3	ODC Ornithine decarboxylase (Capsicum annuum OX=4072)	Polyamine metabolism
SLA-04_05571-RA	3.35	SIS8 Probable serine/threonine-protein kinase SIS8 (Arabidopsis thaliana OX=3702)	Enzyme classification
SLA-04_07041-RA	3.35	ANK1 Ankyrin-1 (Homo sapiens OX=9606)	not assigned
SLA-04_07228-RA	3.35	omh1 O-glycoside alpha-1,2-mannosyltransferase omh1 (Schizosaccharomyces pombe (strain 972 / ATCC 24843) OX=284812)	not assigned
SLA-04_05947-RA	3.35	PHT4;4 Ascorbate transporter, chloroplastic (Arabidopsis thaliana OX=3702)	Solute transport
SLA-04_02239-RA	3.35	PRSS12 Neurotrypsin (Pongo pygmaeus OX=9600)	not assigned
SLA-04_07012-RA	3.35	SKN1 Beta-glucan synthesis-associated protein SKN1 (Candida albicans OX=5476)	not assigned
SLA-04_08345-RA	3.4	TUB Tubby protein homolog (Homo sapiens OX=9606)	RNA biosynthesis
SLA-04_03261-RA	3.45	GATA12 GATA transcription factor 12 (Arabidopsis thaliana OX=3702)	RNA biosynthesis
SLA-04_03294-RA	3.5	PIN1A Auxin efflux carrier component 1a (Oryza sativa subsp. japonica OX=39947)	not assigned
SLA-04_03506-RA	3.55	FAD4L1 Fatty acid desaturase 4-like 1, chloroplastic (Arabidopsis thaliana OX=3702)	Lipid metabolism
SLA-04_02334-RA	3.55	MYB44 Transcription factor MYB44 (Arabidopsis thaliana OX=3702)	RNA biosynthesis

SLA-04_02077-RA	3.55	proB Glutamate 5-kinase (Acaryochloris marina (strain MBIC 11017) OX=329726)	not assigned
SLA-04_00858-RB	3.55	SEC Probable UDP-N-acetylglucosamine--peptide N-acetylglucosaminyltransferase SEC (Arabidopsis thaliana OX=3702)	not assigned
SLA-04_07931-RA	3.65	lrrc40 Leucine-rich repeat-containing protein 40 (Xenopus tropicalis OX=8364)	not assigned
SLA-04_03770-RA	3.65	SYNPCC7002_A0175 Uncharacterized protein SYNPCC7002_A0175 (Synechococcus sp. (strain ATCC 27264 / PCC 7002 / PR-6) OX=32049)	not assigned
SLA-04_06256-RA	3.85	FAM210B Protein FAM210B, mitochondrial (Homo sapiens OX=9606)	not assigned
SLA-04_08149-RA	3.9	Bckdk [3-methyl-2-oxobutanoate dehydrogenase [lipoamide]] kinase, mitochondrial (Mus musculus OX=10090)	Cellular respiration
SLA-04_06486-RA	3.9	At3g61320 UPF0187 protein At3g61320, chloroplastic (Arabidopsis thaliana OX=3702)	Solute transport
SLA-04_00152-RA	3.9	CAS Calcium sensing receptor, chloroplastic (Arabidopsis thaliana OX=3702)	not assigned
SLA-04_01995-RA	3.95	D-galactoside-specific lectin (Heliocidaris crassispina OX=1043166)	not assigned
SLA-04_02671-RA	3.95	PSBS2 Photosystem II protein PSBS2 (Chlamydomonas reinhardtii OX=3055)	not assigned
SLA-04_01706-RA	3.95	SGT1 Peptidyl serine alpha-galactosyltransferase (Chlamydomonas reinhardtii OX=3055)	Protein modification
SLA-04_07847-RA	4.05	At3g61320 UPF0187 protein At3g61320, chloroplastic (Arabidopsis thaliana OX=3702)	Solute transport
SLA-04_01065-RA	4.05	MLO11 MLO-like protein 11 (Arabidopsis thaliana OX=3702)	not assigned
SLA-04_05419-RA	4.05	PF13_0198 Reticulocyte-binding protein 2 homolog a (Plasmodium falciparum (isolate 3D7) OX=36329)	not assigned
SLA-04_07424-RA	4.05	PSR2 Probable phosphatase PSR2 (Saccharomyces cerevisiae (strain ATCC 204508 / S288c) OX=559292)	Lipid metabolism
SLA-04_00319-RA	4.1	CG15309 Protein yippee-like CG15309 (Drosophila melanogaster OX=7227)	not assigned
SLA-04_01154-RB	4.15	ELI3 Probable mannitol dehydrogenase (Mesembryanthemum crystallinum OX=3544)	Enzyme classification
SLA-04_07047-RA	4.2	RPS20 40S ribosomal protein S20 (Macaca fascicularis OX=9541)	Protein biosynthesis
SLA-04_01692-RA	4.2	CBR Carotene biosynthesis-related protein CBR, chloroplastic (Dunaliella salina OX=3046)	Photosynthesis
SLA-04_01238-RA	4.2	Extracellular ribonuclease LE (Solanum lycopersicum OX=4081)	RNA processing
SLA-04_03923-RA	4.25	Os01g0917400 Zinc finger CCCH domain-containing protein 12 (Oryza sativa subsp. japonica OX=39947)	RNA processing
SLA-04_07960-RB	4.25	PDC2 Pyruvate decarboxylase 2 (Nicotiana tabacum OX=4097)	Enzyme classification
SLA-04_00807-RA	4.3	At3g06270 Probable protein phosphatase 2C 35 (Arabidopsis thaliana OX=3702)	not assigned
SLA-04_03192-RA	4.4	SERINC1 Serine incorporator 1 (Bos taurus OX=9913)	sphingolipid
SLA-04_07960-RA	4.5	PDC2 Pyruvate decarboxylase 2 (Fragment) (Pisum sativum OX=3888)	not assigned
SLA-04_05494-RA	4.55	COL5 Zinc finger protein CONSTANS-LIKE 5 (Arabidopsis thaliana OX=3702)	not assigned
SLA-04_04861-RA	4.55	ORLIKE Protein ORANGE-LIKE, chloroplastic (Arabidopsis thaliana OX=3702)	not assigned
SLA-04_02291-RA	4.6	Desiccation-related protein PCC13-62 (Craterostigma plantagineum OX=4153)	not assigned
SLA-04_00955-RA	4.65	BOR2 Probable boron transporter 2 (Arabidopsis thaliana OX=3702)	Solute transport
SLA-04_05164-RA	4.7	glcA Glucan endo-1,3-beta-glucosidase A1 (Bacillus circulans OX=1397)	not assigned
SLA-04_01626-RA	4.75	At2g45870 UPF0187 protein At2g45870, chloroplastic (Arabidopsis thaliana OX=3702)	Solute transport
SLA-04_00878-RB	4.8	PD_1893 UPF0394 membrane protein PD_1893 (Xylella fastidiosa (strain Temecula1 / ATCC 700964) OX=183190)	not assigned
SLA-04_00220-RA	4.85	DCL1 Endoribonuclease Dicer homolog 1 (Arabidopsis thaliana OX=3702)	not assigned
SLA-04_06355-RA	4.9	Barwin (Hordeum vulgare OX=4513)	not assigned
SLA-04_01309-RA	4.95	bgaB Beta-galactosidase bgaB (Geobacillus kaustophilus OX=1462)	not assigned

SLA-04_08116-RA	5	Ankrd6 Ankyrin repeat domain-containing protein 6 (Mus musculus OX=10090)	not assigned
SLA-04_05329-RA	5	Os09g0459600 Probable protein phosphatase 2C 69 (Oryza sativa subsp. japonica OX=39947)	not assigned
SLA-04_01632-RA	5.15	At3g61320 UPF0187 protein At3g61320, chloroplastic (Arabidopsis thaliana OX=3702)	Solute transport
SLA-04_08082-RA	5.25	At4g11730 Putative ATPase, plasma membrane-like (Arabidopsis thaliana OX=3702)	not assigned
SLA-04_00008-RA	5.35	APK1 Adenylyl-sulfate kinase 1, chloroplastic (Arabidopsis thaliana OX=3702)	Coenzyme metabolism
SLA-04_01455-RB	5.35	NEK8 Serine/threonine-protein kinase Nek8 (Homo sapiens OX=9606)	not assigned
SLA-04_01139-RA	5.35	Thermitase (Thermoactinomyces vulgaris OX=2026)	protein degradation
SLA-04_06004-RA	5.4	CBR Carotene biosynthesis-related protein CBR, chloroplastic (Dunaliella salina OX=3046)	Photosynthesis
SLA-04_06618-RA	5.45	unknown function	Phytohormone action
SLA-04_07383-RA	5.55	FPV245 Putative ankyrin repeat protein FPV245 (Fowlpox virus (strain NVSL) OX=928301)	not assigned
SLA-04_03359-RA	5.55	LYPLA2 Acyl-protein thioesterase 2 (Homo sapiens OX=9606)	not assigned
SLA-04_02472-RA	5.55	PGL3 6-phosphogluconolactonase 3, chloroplastic (Arabidopsis thaliana OX=3702)	not assigned
SLA-04_01126-RA	5.8	ACOT9 Acyl-coenzyme A thioesterase 9, mitochondrial (Homo sapiens OX=9606)	not assigned
SLA-04_07341-RA	5.85	AQP5 Aquaporin-5 (Milnesium tardigradum OX=46460)	not assigned
SLA-04_01081-RA	5.85	ARB_07867 WSC domain-containing protein ARB_07867 (Arthroderma benhamiae (strain ATCC MYA-4681 / CBS 112371) OX=663331)	not assigned
SLA-04_03630-RA	5.85	Desiccation-related protein PCC13-62 (Craterostigma plantagineum OX=4153)	not assigned
SLA-04_02883-RA	5.85	ISA1 Isoamylase 1, chloroplastic (Arabidopsis thaliana OX=3702)	starch degradation
SLA-04_02661-RA	5.9	ABCG22 ABC transporter G family member 22 (Arabidopsis thaliana OX=3702)	Solute transport
SLA-04_04052-RA	5.9	p80 Protein P80 (Dictyostelium discoideum OX=44689)	not assigned
SLA-04_05575-RA	6	Histamine oxidase (Arthrobacter globiformis OX=1665)	Enzyme classification
SLA-04_05576-RA	6.05	AMO Peroxisomal primary amine oxidase (Pichia angusta OX=870730)	not assigned
SLA-04_08124-RA	6.1	CYP55A1 NADP nitrous oxide-forming nitric oxide reductase (Fusarium oxysporum OX=5507)	not assigned
SLA-04_05529-RA	6.1	H3b Histone H3.3 type b (Dictyostelium discoideum OX=44689)	not assigned
SLA-04_04001-RA	6.15	Retrovirus-related Pol polyprotein from transposon TNT 1-94 (Nicotiana tabacum OX=4097)	not assigned
SLA-04_03817-RA	6.2	cdc14ab Dual specificity protein phosphatase CDC14AB (Danio rerio OX=7955)	DNA repair
SLA-04_08183-RA	6.2	Retrovirus-related Pol polyprotein from transposon TNT 1-94 (Nicotiana tabacum OX=4097)	not assigned
SLA-04_05507-RA	6.25	sodA Superoxide dismutase [Mn] (Geobacillus stearothermophilus OX=1422)	not assigned
SLA-04_02931-RA	6.35	NADP-specific glutamate dehydrogenase (Fragment) (Chlorella sorokiniana OX=3076) glutamate to ammonium and Aketoglutarate	Nutrient uptake
SLA-04_07123-RA	6.35	Tpsab1 Tryptase (Rattus norvegicus OX=10116)	protease
SLA-04_05615-RA	6.4	Retrovirus-related Pol polyprotein from transposon TNT 1-94 (Nicotiana tabacum OX=4097)	not assigned
SLA-04_04390-RA	6.5	At2g45870 UPF0187 protein At2g45870, chloroplastic (Arabidopsis thaliana OX=3702)	not assigned
SLA-04_02153-RA	6.6	AKR4C10 Aldo-keto reductase family 4 member C10 (Arabidopsis thaliana OX=3702)	Enzyme classification
SLA-04_04006-RA	6.65	Retrovirus-related Pol polyprotein from transposon TNT 1-94 (Nicotiana tabacum OX=4097)	not assigned
SLA-04_04524-RA	6.7	Retrovirus-related Pol polyprotein from transposon TNT 1-94 (Nicotiana tabacum OX=4097)	not assigned
SLA-04_05461-RA	6.7	PEPR1 Leucine-rich repeat receptor-like protein kinase PEPR1 (Arabidopsis thaliana OX=3702)	not assigned

SLA-04_07724-RB	7.05	CHS2 Chitin synthase 2 (Rhizopus oligosporus OX=4847)	not assigned
SLA-04_02821-RA	7.1	Retrovirus-related Pol polyprotein from transposon TNT 1-94 (Nicotiana tabacum OX=4097)	not assigned
SLA-04_01933-RA	7.15	Retrovirus-related Pol polyprotein from transposon TNT 1-94 (Nicotiana tabacum OX=4097)	not assigned
SLA-04_04004-RA	7.25	Retrovirus-related Pol polyprotein from transposon TNT 1-94 (Nicotiana tabacum OX=4097)	not assigned
SLA-04_04003-RA	7.3	Retrovirus-related Pol polyprotein from transposon TNT 1-94 (Nicotiana tabacum OX=4097)	not assigned
SLA-04_06686-RA	7.35	Retrovirus-related Pol polyprotein from transposon TNT 1-94 (Nicotiana tabacum OX=4097)	not assigned
SLA-04_08297-RA	7.55	Retrovirus-related Pol polyprotein from transposon TNT 1-94 (Nicotiana tabacum OX=4097)	not assigned
SLA-04_04002-RA	7.65	Retrovirus-related Pol polyprotein from transposon TNT 1-94 (Nicotiana tabacum OX=4097)	not assigned
SLA-04_01934-RA	7.75	Retrovirus-related Pol polyprotein from transposon TNT 1-94 (Nicotiana tabacum OX=4097)	not assigned
SLA-04_03998-RA	7.8	Retrovirus-related Pol polyprotein from transposon TNT 1-94 (Nicotiana tabacum OX=4097)	not assigned
SLA-04_05614-RA	8.1	Retrovirus-related Pol polyprotein from transposon TNT 1-94 (Nicotiana tabacum OX=4097)	not assigned
SLA-04_08182-RA	8.2	Retrovirus-related Pol polyprotein from transposon TNT 1-94 (Nicotiana tabacum OX=4097)	not assigned
SLA-04_05613-RA	8.3	Retrovirus-related Pol polyprotein from transposon TNT 1-94 (Nicotiana tabacum OX=4097)	not assigned
SLA-04_01152-RA	8.35	MRS2-7 Magnesium transporter MRS2-7 (Arabidopsis thaliana OX=3702)	Solute transport
SLA-04_08298-RA	8.35	Retrovirus-related Pol polyprotein from transposon TNT 1-94 (Nicotiana tabacum OX=4097)	not assigned
SLA-04_04005-RA	8.45	Retrovirus-related Pol polyprotein from transposon TNT 1-94 (Nicotiana tabacum OX=4097)	not assigned
SLA-04_02822-RA	8.8	Retrovirus-related Pol polyprotein from transposon TNT 1-94 (Nicotiana tabacum OX=4097)	not assigned
SLA-04_06502-RA	8.9	sll1483 Uncharacterized protein sll1483 (Synechocystis sp. (strain PCC 6803 / Kazusa) OX=1111708)	Cell wall modification
SLA-04_03453-RA	9	pho-4 Phosphate-repressible phosphate permease pho-4 (Neurospora crassa (strain ATCC 24698 / 74-OR23-1A / CBS 708.71 / DSM 1257 / FGSC 987) OX=36	Solute transport
SLA-04_00239-RA	9.4	ACA11 Putative calcium-transporting ATPase 11, plasma membrane-type (Arabidopsis thaliana OX=3702)	not assigned
SLA-04_05577-RA	9.45	AMT1-2 Ammonium transporter 1 member 2 (Arabidopsis thaliana OX=3702)	not assigned
SLA-04_08127-RA	9.45	GLOX Aldehyde oxidase GLOX (Vitis pseudoreticulata OX=231512)	not assigned
SLA-04_03639-RA	9.95	SLT1 Sodium/sulfate cotransporter 1 (Chlamydomonas reinhardtii OX=3055)	not assigned
SLA-04_03358-RA	13	sll1483 Uncharacterized protein sll1483 (Synechocystis sp. (strain PCC 6803 / Kazusa) OX=1111708)	Cell wall modification
